# Disentangling the Three Facets of Mass Ideological Polarization: A Network Approach Across 78 Societies

**DOI:** 10.1093/poq/nfag037

**Published:** 2026-09-08

**Authors:** Yufan Guo, Yilang Peng, Tian Yang

**Affiliations:** PhD Student, School of Journalism and Communication, The Chinese University of Hong Kong, Shatin, Hong Kong, China; Associate Professor, Department of Financial Planning, Housing and Consumer Economics, University of Georgia, Athens, GA, US; Assistant Professor, School of Journalism and Communication, The Chinese University of Hong Kong, Shatin, Hong Kong, China

## Abstract

Public opinion research has intensively examined mass ideological polarization, often linking it to outcomes that threaten key democratic ideals. Both theory and empirical evidence suggest that it comprises multiple manifestations, with their relationships, however, remaining largely unclear. Meanwhile, previous studies have seldom conducted cross-contextual comparisons, which are essential for understanding how macrostructural factors shape mass polarization. These limitations partly arise from the methodological challenges that hinder consistent measurement across diverse contexts. To fill these gaps, this study introduces a belief network approach to compare three core manifestations of mass ideological polarization: disagreement, symbolic-operational ideological alignment, and within-operational ideological alignment. Using data from 78 societies in the World Values Survey and the European Values Study (2017–2023), we employ improved network-based measures that capture ideological alignment without relying on context-specific assumptions. Surprisingly, our findings reveal substantial cross-national variation and contrasting correlations among these manifestations, indicating that mass ideological polarization is neither singular nor universal. We further apply the network approach to examine how modernization, a critical macrostructural factor, shapes ideological polarization, showing that it predicts lower disagreement but higher ideological alignment. By offering a scalable and context-adaptable tool for comparative public opinion research, this study proposes an expanded theoretical framework of mass polarization, highlighting the need to distinguish its diverse manifestations and their structural drivers across global contexts.

Mass ideological polarization has become a critical challenge for many societies worldwide and has prompted scholarly efforts to assess its scope and manifestations (Lelkes 2016; Silver 2022). Some scholars have highlighted the multidimensional nature of mass ideological polarization, viewing it as the opinion divergence on specific issues among the whole public or as the consistency between various issue stances and their association with political identities (DiMaggio et al. 1996; Fiorina and Abrams 2008; Hetherington 2009; Lelkes 2016; Jost et al. 2022). With its various manifestations, mass ideological polarization carries profound normative consequences: It entrenches rigid partisan worldviews, amplifies extremism at the expense of moderate consensus, undermines democratic compromise by substituting tribal loyalty for policy deliberation, and fosters societal fragmentation (McCoy et al. 2018; Gollwitzer et al. 2020).

Despite this conceptual breadth, extensive empirical research either conflates these manifestations or focuses narrowly on just one in the name of polarization (see Mehlhaff 2024 for a review). Such approaches rest on an implicit assumption that these manifestations co-occur and move in tandem. However, the empirical relationships among them remain unclear, raising fundamental questions about whether ideological polarization is a unified process or a set of distinct, independent dynamics. This ambiguity largely stems from the lack of cross-contextual comparisons, as existing research predominantly focuses on the longitudinal trends of a single or a few Western democracies (Perrett 2021; Merkley 2023), especially the United States (Baldassarri and Gelman 2008; Park 2018; DellaPosta 2020; Kozlowski and Murphy 2021). Such a narrow empirical scope obscures how ideological polarization unfolds in other contexts, which limits our ability to characterize context-specific configurations that may diverge from general patterns (Matassi and Boczkowski 2023). It also impedes investigations into how macro-structural factors, often connected deeply to contexts, shape ideological polarization.

We argue that these gaps may partially result from a critical methodological constraint: the absence of systematic approaches to quantify and compare ideological polarization across societies. Given the substantial variations in political cultures and societal environments, generating cross-contextually comparable measures of mass ideological polarization, especially for each specific manifestation, is inherently difficult (Mehlhaff 2025). Measurement strategies developed in one context, such as tracking partisan identity alignment, or issue alignment across economic (e.g., government welfare) and social domains (e.g., gay rights) in the United States, may not sufficiently capture ideology structures in other party systems and political cultures (Zechmeister and Corral 2013; Pan and Xu 2018).

In response, we introduce a belief network-based approach to operationalize three theoretically distinct manifestations of mass ideological polarization in a cross-contextually comparable framework: (1) *disagreement*, the extent of attitudinal divergence; (2) *within-operational ideological alignment*, the degree of ideological coherence in individuals’ belief systems; and (3) *symbolic-operational ideological alignment*, the degree of sorting between symbolic ideology and operational ideology. Drawing on data from the World Values Survey (WVS) and the European Values Study (EVS) from 2017–2023 in 78 societies, we measure these manifestations on a global scale, test how these three are interrelated across global contexts, and investigate how macrostructural factors predict each manifestation.

This comparative study, therefore, makes several contributions to polarization scholarship. Theoretically, we clarify conceptual dimensions of mass ideological polarization and propose how they manifest differently across social contexts. Methodologically, we refine network-based measures to operationalize ideological alignment in a more accurate way, which enables systematic cross-national comparisons. Empirically, we establish a global ranking of societies on these polarization manifestations, revealing significant cross-contextual variations. We demonstrate that these manifestations do not always evolve in tandem, which provides evidence that mass ideological polarization should be better analyzed as a multifaceted construct. Finally, we test how modernization distinctively predicts each manifestation and uncover how previously overlooked macrostructural factors shape ideological polarization.

## Mass Ideological Polarization: A Multidimensional Concept

Polarization, as an important social science concept, has been broadly used to refer to a wide range of social dynamics, including affective feelings toward outgroup members (i.e., affective polarization; Iyengar et al. 2012), perception about intergroup animosity (i.e., perceived polarization; Bail 2022), as well as elite polarization between political leaders (Barberá et al. 2015). This study focuses on mass ideological polarization, which is primarily driven by ideology-based divisions within the mass public (Lelkes 2016). Here, we define political ideology as a “set of beliefs about the proper order of society and how it can be achieved” (Erikson and Tedin 2015, 70). Public opinion scholars usually distinguish between *symbolic* and *operational* elements of political ideology (Jost et al. 2009; Federico and Malka 2023). Symbolic ideology refers to the identity-based element reflecting individuals’ self-identification with broad ideological labels, such as liberal or conservative, left or right (Ellis and Stimson 2012; Mason 2018). Operational ideology, in contrast, covers the issue-based element that reflects actual political values, policy preferences, and concrete issue positions (Converse 2006; Ellis and Stimson 2012). Accordingly, ideological polarization also involves both elements: polarization based on their symbolic attachments to ideological identities, and polarization in specific ideological preferences.

**Figure 1.**
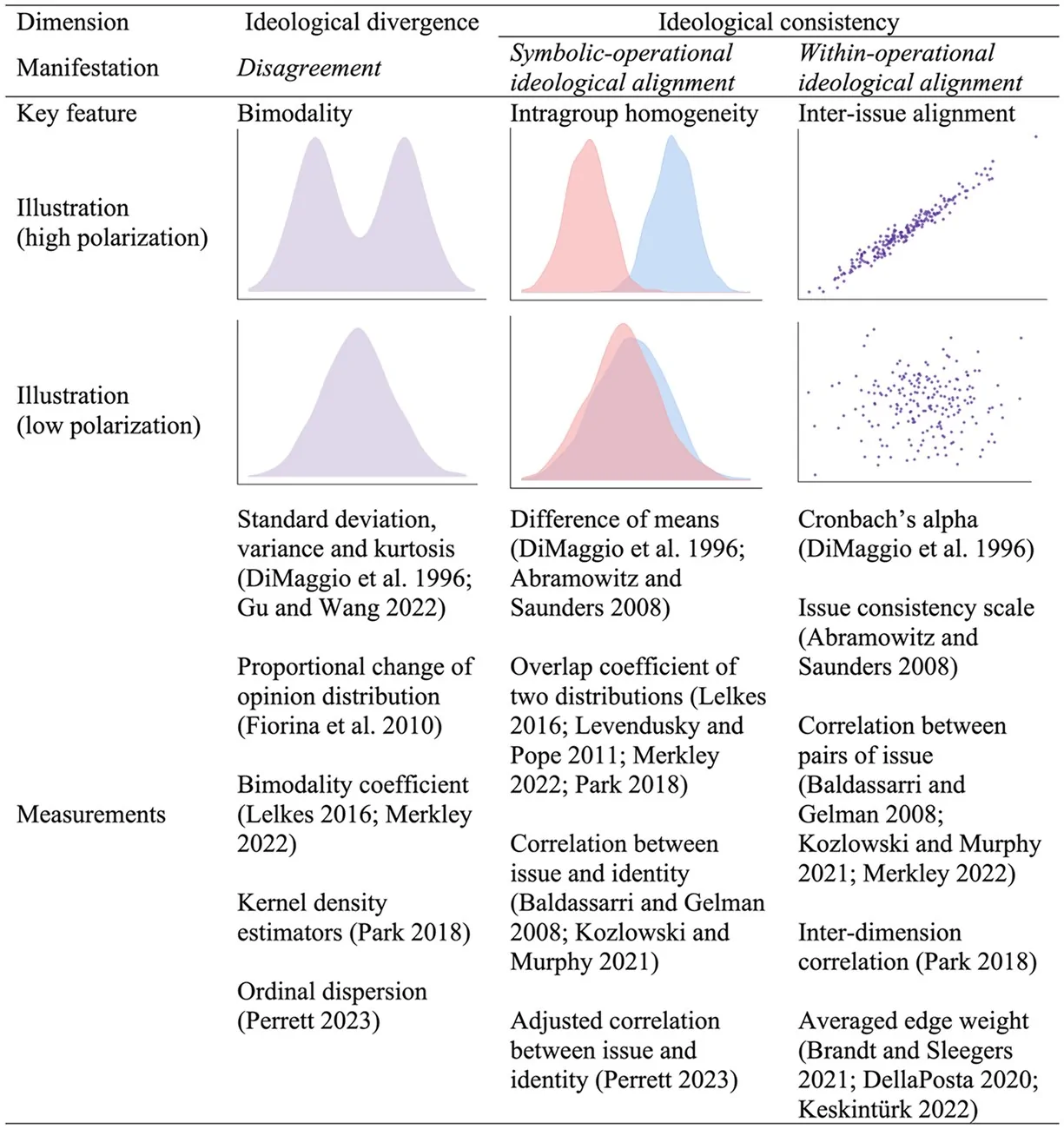
Summary of three manifestations of mass ideological polarization.

Indeed, mass ideological polarization has long been recognized as a multidimensional phenomenon, capturing different distributional features of public opinion. In their key work, DiMaggio et al. (1996) outlined two sets of strategies that characterize mass ideological polarization. The first focuses on properties of a single distribution, namely, the extent to which public opinion is heterogeneous. The second examines relational properties among distributions, particularly the homogeneity within different ideological or attitudinal clusters. These two separate conceptual dimensions, formally described as ideological divergence and consistency (Lelkes 2016), have continued to shape the theorization of mass ideological polarization in subsequent literature (e.g., Fiorina and Abrams 2008; Lelkes 2016; DellaPosta 2020; Mehlhaff 2024). We summarize their major manifestations and common measurements in figure 1.

Corresponding to the ideological divergence dimension, *disagreement* refers to the extent of ideological opinion dispersion within a certain public (DiMaggio et al. 1996). It assesses how far the population is spread out along a certain opinion spectrum, with higher levels indicating increased extremism at both ends and a declining presence of centrist views. As distributions shift from unimodal (centered) to bimodal (polarized), the public becomes more divided (Fiorina and Abrams 2008). Common empirical strategies to measure disagreement vary from simple variance, kurtosis (DiMaggio et al. 1996), and the proportion of extreme views (Fiorina et al. 2010), to more complicated indices like the bimodality coefficient (Lelkes 2016; Merkley 2023).

The ideological consistency dimension, however, has two manifestations. *Symbolic-operational ideological alignment* refers to the degree of alignment between individuals’ operational ideological positions and their symbolic ideological identities. This resembles the influential notion of partisan sorting (Levendusky 2009), which assesses the alignment between party identification and issue positions, and was regarded as an important reason to explain the observed increased polarization among the American public (Fiorina and Abrams 2008; Lelkes 2016). In a well-sorted mass public, partisan and issue-specific preferences are internally cohesive. For example, Democrats in the United States tend to support environmental protection, while Republicans are more likely to oppose gun control. This identity-issue alignment suggests that people’s attitudes are somewhat homogeneous within each political identity group (Mehlhaff 2024). To generalize this idea beyond the two-party context, we focus here on an alternative form of “sorting” driven by symbolic ideology as a political identity, namely, the extent to which individuals’ ideological identifications are aligned with their attitudes on concrete values and issues. Importantly, while disagreement can exist without systematic alignment to partisan or ideological identity, ideological alignment is often limited in contexts where disagreement is minimal (Perrett 2023). Sorting (more generally, symbolic-operational ideological alignment) was typically measured by the correlations between partisan (and ideological) identification and specific issue positions (Baldassarri and Gelman 2008; Kozlowski and Murphy 2021).

The other manifestation of ideological consistency is *within-operational ideological alignment*, which evaluates whether multiple operational ideological stances (i.e., values, preferences, issue positions) are interrelated and bound together. It can be traced to Converse’s (2006) work on “constraint,” which means that an individual who holds certain beliefs also holds other beliefs. Unlike sorting that reflects identity-based polarization, a population’s within-operational ideological alignment indicates how often certain pairs of specific opinions co-occur (Baldassarri and Gelman 2008; Kozlowski and Murphy 2021). For example, US citizens who support environmental protection may also support LGBTQ+ and abortion rights. High within-operational ideological alignment indicates that individuals hold coherent beliefs that can be predicted by certain underlying ideological principles, while a low value reflects issue-specific opinions and a loose organization of belief system (Converse 2006). Scholars usually measure within-operational ideological alignment using the multi-issue consistency scale (Abramowitz and Saunders 2008) or the correlation between pairs of issues (Baldassarri and Gelman 2008; Kozlowski and Murphy 2021).

While these measures have offered rich insights, several challenges remain when attempting to generalize existing findings to broader contexts. First, many studies that claim to examine polarization focus empirically on only a single manifestation. According to Mehlhaff’s (2024) review, the majority of polarization literature relies solely on simple metrics like means or standard deviations. This conceptual and empirical imbalance limits the exploration of a key question: Do the three manifestations of ideological polarization unfold together or operate independently? If the latter, it becomes crucial to examine how they are shaped by different dynamics. Second, most multidimensional analyses are situated within the US context. Although a few studies have extended to the Canadian (Merkley 2023) or British public (Perrett 2021), we still know little about ideological polarization in the global context, especially in the Global South. The lack of comparative studies prevents us from understanding how macrostructural factors explain polarization patterns.

## Limitations of Mass Ideological Polarization Measures and the Belief Network Approach

These theoretical gaps partly arise from several interrelated methodological concerns that hinder applying context-specific measurements to other societies. First, many existing measures adopt an overly narrow perspective on public opinion. Some rely solely on a single liberal-conservative spectrum (Dalton 2006), while others concentrate exclusively on a handful of issue domains (Fishman and Davis 2022; Rigoli 2025; for an exception, see Perrett 2023). This constrained approach could fail to reflect the diversity and breadth of political views within societies and often falls short in cross-contextual comparisons, where political divisions and salient issues may differ substantially.

Second, some measures assume a fixed ideological structure, typically based on Western contexts. For example, Park (2018) evaluated issue alignment in the United States by categorizing issue attitudes into three ideological dimensions: economic, civil rights, and moral issues. But such a fixed structure may not map neatly onto other political cultures (Dalton 2006; Malka et al. 2019). Any comparison of mass ideological polarization, therefore, requires context-adaptable analytical means. Indeed, only a few studies have attempted to develop standardized tools for cross-national measurement, such as expert-based rating (Coppedge et al. 2025) and composite indices (Mehlhaff 2025). Still, while helpful in generating broad comparisons, these efforts often reduce polarization to a single summary measure.

Third, although relational measures are increasingly popular for considering multiple opinion items, most strategies risk overestimating the strength of connections, leading to inflated estimates of ideological polarization. One type of relational measures relies on the belief network, where each node represents an individual belief item, and each edge denotes the relationship between the connected belief items (e.g., how strongly two beliefs are correlated within a public). For instance, Boutyline and Vaisey (2017) constructed a network consisting of multiple nodes representing various belief items and one node indicating the ideological identity, with ties representing pairwise correlations between them. They found that the ideological identity in the United States has the largest betweenness centrality, which indicates its central role in shaping other items within belief systems. This network analysis, which leverages information from all pairs of belief items, helps identify structural features such as the central and peripheral positions of beliefs (for other applications, see Brandt et al. 2019; Fishman and Davis 2022). Thus, one of the belief network’s advantages is that it allows for an expanded size of belief items without imposing any predefined structure, thus facilitating cross-contextual comparability.

An important choice in constructing belief networks is choosing between simple correlations and partial correlations. Many existing applications rely on simple correlations between pairs of belief items to gauge the strength of their connections. Yet these pairwise correlations capture both direct and indirect associations: The observed correlation between two items may be largely driven by their associations with a third item. In contrast, partial correlations isolate the unique relationship between two items by controlling for all others, and provide a more accurate representation of the underlying network structure (Epskamp and Fried 2018; Borsboom et al. 2021). In the context of belief network, failing to distinguish between spurious and direct connections may make belief systems appear more tightly integrated than they actually are.

**Figure 2.**
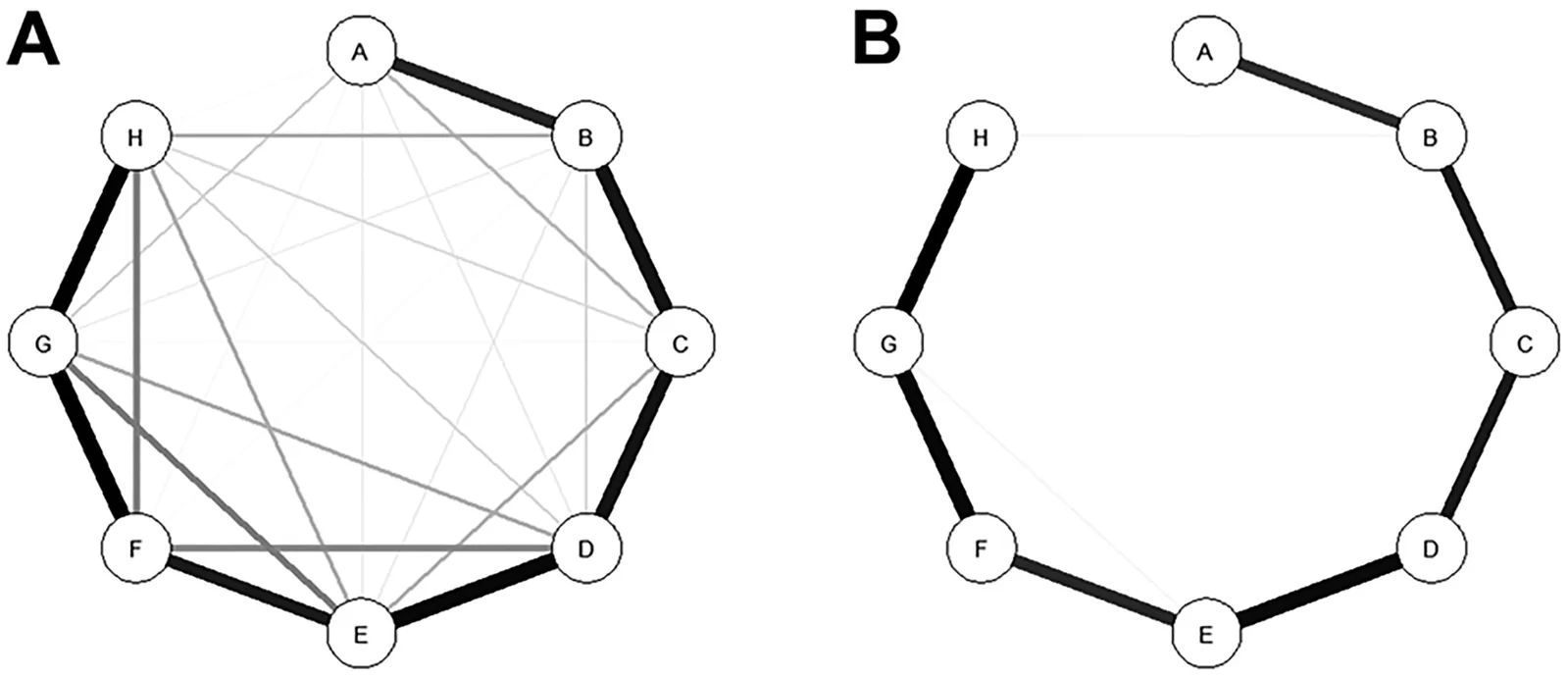
Correlation-based vs. partial correlation network from simulated data. In both panels, the thickness of edges corresponds to the magnitude of the association between nodes. We generated data for 1,000 simulated individuals on eight attitudinal variables (A–H) under a prespecified model, where only adjacent items were conditionally dependent (e.g., A–B, B–C, C–D, and so forth) after controlling for all other variables. All direct associations between adjacent nodes were set up as 0.3, and no direct association exists between nonadjacent nodes. Panel A shows the network constructed from pairwise correlations among variables A–H. Those extra edges between nonadjacent nodes (e.g., A–C, E–G) represent spurious, indirect associations mediated through other variables. Panel B shows the network based on partial correlations, where edges represent conditional dependencies controlling for all other variables. Only adjacent variables are connected, and edges here are much sparser, showing only direct associations specified in the data-generating process. See Supplementary Material section H for more details.

We use a simple simulation to illustrate this problem (figure 2). In a situation where all direct associations between adjacent nodes were set up as 0.3, and no direct association exists between nonadjacent nodes, simple correlations (Panel A, with extra indirect associations identified) will overestimate the density and strength of connections compared to partial correlations (Panel B).

To address these limitations, our study adopts two key methodological strategies. First, we move beyond the US-based ideological structure, incorporating a wide range of over 30 ideological items drawn from public opinion surveys. This approach allows us to capture diverse political beliefs across different societies without making prior assumptions about which items should be considered more important or politically salient. Second, we leverage a conditional dependency network approach that reduces the aforementioned indirect associations to analyze ideological alignment. This method enables us to more accurately reveal the actual ideological structures.

## Three Manifestations of Mass Ideological Polarization Across the Globe

Understanding ideological polarization requires a global comparative perspective, as its manifestations may vary substantially across societies. Existing literature on polarization often treats it as a singular phenomenon, yet the few cross-contextual comparisons reveal a more multifaceted reality. A society may rank high on one indicator of polarization but low on another. For instance, Mehlhaff’s (2025) data ranked India as less ideologically polarized than Australia in 2019, yet V-Dem classified India as experiencing “Serious polarization” and Australia as only “Medium polarization” (Coppedge et al. 2025). These inconsistencies may stem not only from measurement challenges. Instead, they point to the more fundamental challenge surrounding the conceptual clarity and alignment of various manifestations of ideological polarization. The application of network measures, in this case, enables us to investigate three manifestations of ideological polarization across societies. We thus ask:

**RQ1:** What is the global distribution of societies across the three manifestations of mass ideological polarization?

Furthermore, despite the intuitive assumption that these polarization manifestations should move together, the lack of comparative studies prevents us from obtaining a clear answer. Indirect empirical evidence, mostly derived from longitudinal studies based on a single context, has shown mixed findings. Longitudinal evidence from American public opinion shows that partisan sorting has continued to rise in the past few decades, even in the absence of increased ideological divergence (DiMaggio et al. 1996; Jocker et al. 2024) or within-operational ideological alignment (Baldassarri and Gelman 2008). Meanwhile, recent evidence suggests that within-operational ideological alignment may be catching up (Kozlowski and Murphy 2021), and disagreement seems to be stable over time (Perrett 2023). In Canada, Merkley (2023) documents modest divergence but increasing levels of both partisan sorting and within-operational ideological alignment. These complex and sometimes contradictory patterns suggest that ideological polarization may not be a singular process, but a constellation of related yet independent dynamics. We thus formally raise this question at the global scale:

**RQ2:** What are the relationships among the three manifestations of mass ideological polarization across societies?

## Application: How Modernization Predicts Mass Ideological Polarization

One critical advantage of comparative studies is to illuminate the role of macrostructural factors in shaping mass polarization. Here, we apply our network-based, multidimensional measures to investigate a key factor that involves a crucial theoretical debate: whether modernization fosters or restrains ideological polarization. Modernization theories tend to view economic, political, and cultural change as a holistic process, often occurring in tandem and driving the overall value shift toward more liberal and postmaterialist values (Inglehart and Welzel 2005). However, they did not fully consider the evolution of value variability within societies. While previous studies have primarily focused on disagreement (Dalton 2006; Rigoli 2025), we look at how modernization influences all three manifestations.

When examining disagreement, two competing explanations point to contrasting roles of modernization (Rigoli 2025). The Social Complexity Model views modernization as a catalyst for increasing ideological conflict. As societies develop, traditional norms are disrupted (Giddens 1990), and individualization, urbanization, and lifestyle diversification create an affluent marketplace of ideas (Divale and Seda 2001). Societies thus become more differentiated across occupational, cultural, and informational domains, generating new axes of identity and belief (Durkheim 1997; Inglehart and Welzel 2005). This differentiation lays the groundwork for the emergence of a broader spectrum of ideologies, increasing the possibility of people dividing into distinct opinion groups.

In contrast, the Social Transition Model suggests that ideological conflict, as a symptom of social transformation, should decrease as societies develop (Bell 1960). As modernity consolidates, economic growth, technological diffusion, and institutional stability promote greater social consensus, especially on those economic issues related to survival needs (e.g., food and security). Empirically, this model is supported by evidence showing less political extremism (Dalton 2006; Rigoli 2023) and fewer dispersed opinions (Rigoli 2025) in more developed societies.

Modernization’s influence on ideological alignment, however, may operate through different mechanisms. One is ideological institutionalization. As traditional sources of social cohesion (e.g., religion or local communities) erode, political ideology increasingly becomes a central axis for managing social conflict and organizing collective identity (Grofman 2004). In advanced industrial democracies, this shift is accompanied by the rise of institutionalized party systems (Mainwaring and Torcal 2006), where political parties act as “cognitive authorities” (Keskintürk 2022). They exhibit coherent ideological profiles, maintain strong connections with their social bases, and develop durable organizational infrastructures (Keskintürk 2022). These party structures usually offer clear identity cues to the electorate, enabling them to sort themselves along partisan lines and to navigate an increasingly diverse ideological space (Hetherington 2001; Robison and Mullinix 2016; Merkley 2023). Meanwhile, modernization is often accompanied by a rise in the public’s political interest (Inglehart and Welzel 2005; Hu and Solt 2025), enabling citizens to acquire more political knowledge and to better understand and conform to specific ideological orientations. When institutionalized political ideology encounters a politically interested public, they jointly give rise to a more cohesive and tightly structured ideological system. And political ideology itself increasingly becomes a social identity (Ellis and Stimson 2012; Mason 2018), strengthening symbolic cleavages and tightening issue alignment. In contrast, less developed societies tend to exhibit more ideologically innocent citizens (Mainwaring and Torcal 2006).

Taken together, these theoretical arguments lead us to the following question:

**RQ3:** How does modernization predict the three manifestations of mass ideological polarization?

## Methods

### Data

This study relies on the joint EVS and WVS 2017–2022 dataset, which includes responses from 156,658 individuals across 92 societies (EVS/WVS 2022). The questionnaire covers a broad array of attitudes and beliefs in various issue domains, making it the most comprehensive and globally inclusive dataset available for comparative public opinion research. See more dataset details in Appendix A.

To compile our list of ideological items, we began with the typical bidimensional framework of operational ideology: economic and social dimensions (Jost et al. 2009). Yet, this structure is largely derived from US-based evidence (Carmines et al. 2012; Feldman and Johnston 2014). In many Western democracies, support for liberal democracy over alternative regime types has traditionally been taken for granted. But this “consensus” does not necessarily hold in other societies, where support for different governance types constitutes an important debate in public political preferences (Zechmeister and Corral 2013; Pan and Xu 2018). And recent research documents signs of declining support for democratic principles even within established democracies (Claassen and Magalhães 2023; Foa and Mounk 2025). To avoid imposing a Western-centric conception of ideology on other regions, we included additional ideological items beyond social and economic issues to capture preferences for different types of government and understandings of democracy. Based on this operationalization, we systematically reviewed the questionnaire, and selected operational ideological items that reflect a value, belief, or preference in the economic, social, and governmental/democratic domains. We also referenced the item list from Keskintürk (2022) and made several additions and removals to suit our analytical aims. Considering the potential of item-level nonresponse that could reduce the number of societies in analysis, we finally retained 34 ideology-related items. These items span a range of values and issues: The economic dimension includes issues such as social welfare, economic inequality, and environmental protection; the social dimension includes authoritarianism, moral traditionalism, death penalty, immigration, and ethnonationalism; and the additional dimension includes mass preferences over government system and democracy. After excluding all cases with missing data, the final analytic sample includes 74,886 individuals from 78 societies. The full lists of selected items and contexts are provided in Supplementary Material section A. We also replicated the analyses on only the social and economic items, suggesting that our findings are not driven by the amalgamation of different item categories (see Supplementary Material section B).

We also complemented the survey data with several society-level indicators from multiple data sources, such as the Worldwide Governance Indicators from the World Bank, the United Nations Development Program (UNDP), and Press Freedom scores from Reporters Without Borders (see Supplementary Material section C). Because survey fieldwork did not always occur in the same year, we matched each respondent to the society-level data corresponding to their specific survey year. We then averaged values across survey years within each society to create society-level variables for analysis. Supplementary Material section D presents a robustness check in which each society-year combination was analyzed separately.

### Measures

**Figure 3.**
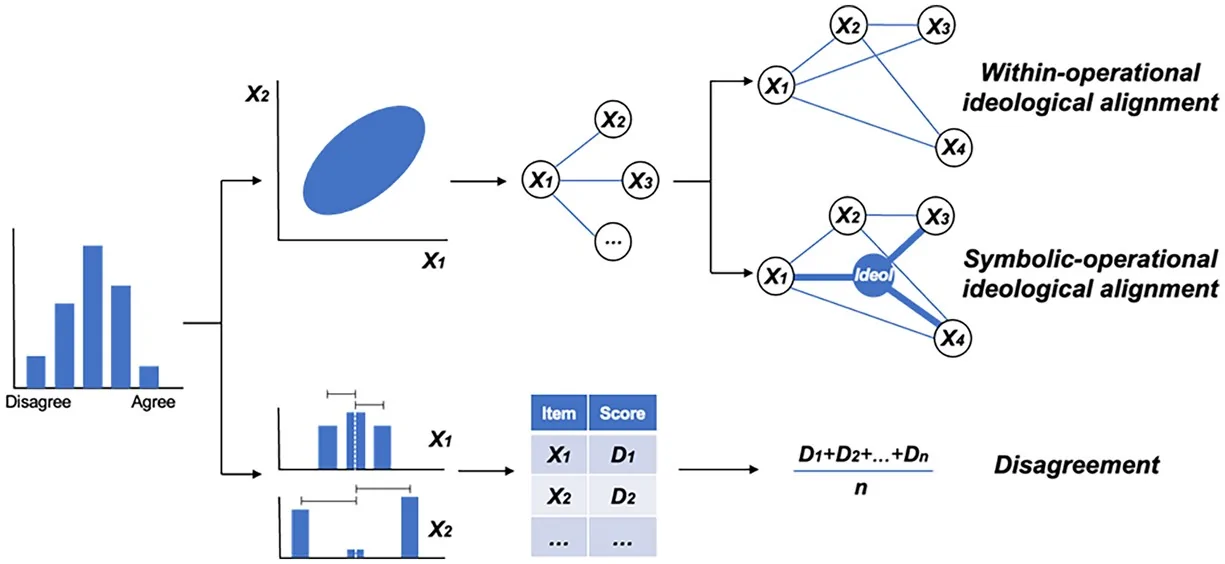
Illustration of our measurements of mass ideological polarization. All our measurements draw on the distributional features of multiple survey items. *X_1_*, *X_2_*, … *X_n_* represent different survey items, which are used to construct two networks: one for measuring within-operational ideological alignment, and another (including an additional IDEOLOGY node) for measuring symbolic-operational ideological alignment. Item-level disagreement scores *D_1_*, *D_2_*, … *D_n_* are computed using the ordinal dispersion equation (Perrett 2023) and then averaged to produce the overall disagreement score.

Figure 3 illustrates our workflow for measuring three manifestations of mass ideological polarization. We validated our three measures by comparing them with alternative measures and other correlated indicators in Supplementary Material section E.

#### Disagreement

We first adopted an item-level measure developed for ordinal scales (Tastle and Wierman 2007; Perrett 2023), using the following equation:


$$D_{j}=-\sum_{k=1}^{n}P(j_{k})\log_{2}(1-\frac{\left|j_{k}-\mu_{j}\right|}{d_{j}})$$

Here, *j* is an ordered response variable (i.e., one survey item) with *n* observations and *K* possible values $j_{1},\;j_{2},\ldots ,\;j_{k}$. $\mu_{j}$ is the mean response to item *j*, and $d_{j}$ is the maximum possible distance between these values. This yields a value between 0 and 1, with higher scores indicating greater opinion dispersion around the item’s mean response. We calculated overall disagreement by averaging disagreement scores across 34 items. Compared to traditional variance-based measures, this measure accounts for both the spread and extremity of opinion distributions on an ordinal scale. This average disagreement value, which revealed strong positive correlations with disagreement values of most items (see Supplementary Material  table A1), should reflect a general trend rather than being driven by a few items.

**Table 1.** The top 10 societies in each manifestation.

Rank	Disagreement	Symbolic-operational ideological alignment	Within-operational ideological alignment
1	Ethiopia (0.59)	Switzerland (1.33)	Canada (14.97)
2	Nicaragua (0.56)	Iceland (1.15)	Great Britain (13.24)
3	Colombia (0.56)	Canada (1.14)	Morocco (13.02)
4	Puerto Rico (0.56)	Great Britain (1.11)	United States (12.89)
5	Montenegro (0.55)	Australia (1.03)	Switzerland (12.59)
6	India (0.55)	United States (1.01)	Netherlands (11.21)
7	Kenya (0.53)	Netherlands (0.87)	Australia (11.17)
8	Zimbabwe (0.53)	Italy (0.75)	Germany (11.12)
9	Bosnia and Herzegovina (0.53)	Norway (0.75)	Tunisia (10.89)
10	Guatemala (0.53)	Sweden (0.74)	Serbia (10.74)

*Note:* Score values are indicated in parentheses following society names.

#### Within-operational ideological alignment

To assess how strongly holding one belief predicts holding others, we built a belief network comprising only operational ideology items for each society. In these networks, each node represents a distinct issue stance or preference, and edges represent the partial correlation coefficients between opinion distributions of the two linked nodes, conditional on the influence from all other nodes. The stronger and more numerous these correlations, the more interconnected the beliefs are in the network. Thus, the belief network embodies the structure of within-operational ideological alignment itself: A dense network suggests that beliefs do not operate independently but rather resonate with each other as part of a coherent ideological framework. Conversely, a sparse network suggests low within-operational ideological alignment, where individuals may hold idiosyncratic combinations that are not strongly linked (DellaPosta 2020).

We built a belief network for each society by including 34 items. The network was constructed by using the *bootnet* package (Epskamp et al. 2018), which leverages pairwise Markov random field (PMRF) models, a popular network model widely applied to analyze psychological networks. We specified the estimation approach as “mgm” (i.e., Mixed Graphical Model, see Haslbeck and Waldorp 2020), which allows the incorporation of both binary and continuous variables, enabling us to account for the heterogeneous measurement levels of the included items (Epskamp et al. 2018). Notably, *bootnet* leverages the Least Absolute Shrinkage and Selection Operator (LASSO) penalty, aiming to shrink many small or irrelevant edge weights to exactly zero. This regularization exercise enhances network interpretability and reduces overfitting by retaining only the most meaningful connections.

We used the sum of edge weights within each network as a measure of within-operational ideological alignment (DellaPosta 2020). A network with a higher sum indicates that opinions within the network are more strongly correlated. We also conducted a robustness test using network density, the ratio of the number of edges and the number of possible edges (Wasserman and Faust 1994), as an alternative measure (Fishman and Davis 2022; see Supplementary Material section F).

#### Symbolic-operational ideological alignment

We measured the extent to which a belief system is organized around a central ideological identity by extending the belief network to include both operational ideology nodes and a node representing ideological identity. Since more central nodes are considered more important for binding the entire network (Brandt et al. 2019; Fishman and Davis 2022), symbolic-operational ideological alignment can be characterized by how central the ideological identity node is within the overall network structure. A central position suggests a highly sorted belief system, where ideological identity acts as a structural anchor that shapes the configuration of a wide range of beliefs (Boutyline and Vaisey 2017). In contrast, when the ideological identity node is peripheral or weakly connected with other belief items, the system is less sorted, and beliefs are more likely to be influenced by diverse factors outside of ideological identity.

More specifically, building on our previous network of 34 operational ideology items, we introduced an additional IDEOLOGY node (DellaPosta 2020). It asked respondents’ self-placement on a ten-point left-right ideological scale. Although the precise content of “left” and “right” may vary across national contexts, at least within each society, the left-right continuum consistently reflects a pair of competing ideological orientations (Federico and Malka 2023), regardless of the specific implication of each end. We operationalize symbolic-operational ideological through respondents’ ideological self-identification rather than partisan identification, because the latter’s substantive interpretation varies across institutional contexts, particularly in systems with weakly institutionalized or single-party structures. Moreover, party identification is typically measured as a categorical variable, and cross-national differences in the number and ideological positioning of parties complicate its integration into a belief network. The left-right ideological labels thus offer a practical alternative.

These 35 items together formed a new belief network following the same procedure as before. We then used the in-degree centrality of the IDEOLOGY node, which incorporates both the number of edges and the edge weights (Opsahl et al. 2010), to measure symbolic-operational ideological alignment. In this case, indegree centrality represents the degree to which all political beliefs align with the dominant ideological identity. We did not adopt other common centrality measures for node positions, such as betweenness and closeness centrality. Recent studies have challenged that these measures could not apply to psychological networks (Bringmann et al. 2019; Borsboom et al. 2021). Additionally, both measures can lack stability, complicating their interpretation (Epskamp et al. 2018).

#### Modernization

We used the Human Development Index (HDI) published by UNDP to measure the socioeconomic development level of each society (Inglehart and Welzel 2010; Rigoli 2023). It combines gross national income per capita, life expectancy at birth, and years of education into a composite index (UNDP 2024), thus reflecting the major dimensions of modernization distinguishing societies worldwide. Because GDP per capita is also a common proxy for modernization (Rigoli 2025), we also leveraged this alternative measure in a robustness check (see Supplementary Material section G).

Notably, both sides of theoretical expectations regarding disagreement may yield a more nuanced empirical pattern: Disagreement may initially increase due to growing complexity, but later reduce as it is absorbed by mature modernity. This would imply a curvilinear, inverted U-shaped relationship between disagreement and modernization at different stages. Thus, we included both a linear term and a quadratic term in the regression models.

Besides, we controlled for several structural factors in our analysis, including political (political stability), media (political parallelism and press freedom), and social (total population and ethnic fractionalization) aspects. These factors have been widely used in previous comparative public opinion studies (e.g., Toff and Kalogeropoulos 2020; Keskintürk 2022; Chan and Yi 2024). More detailed clarification, measurements, and descriptive statistics are provided in Supplementary Material section C. Given the different scales of these variables, we reported standardized coefficients in regression analyses.

## Results

### Divergent Distributions Across Three Manifestations of Mass Ideological Polarization

**Figure 4.**
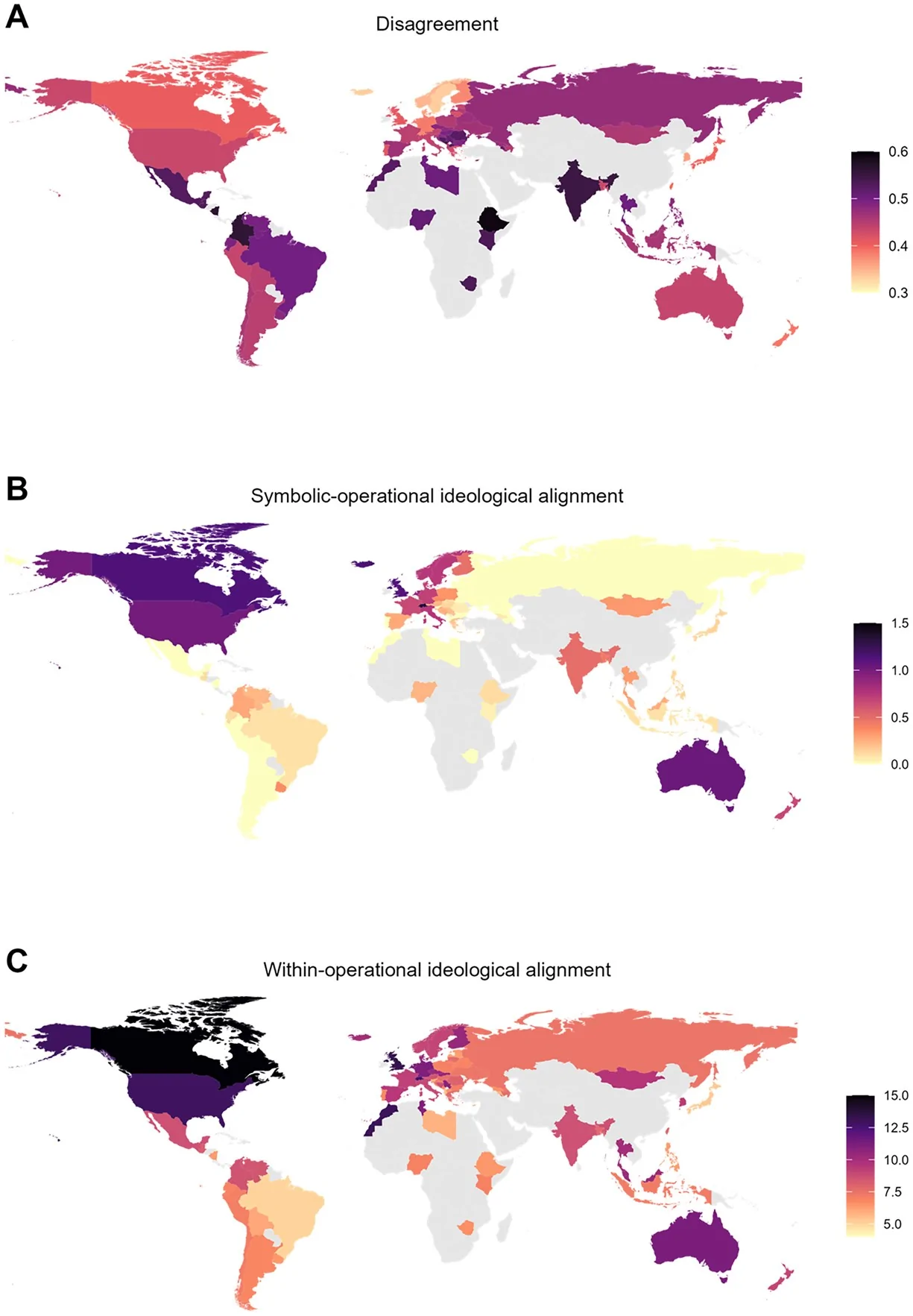
The global distribution of disagreement, symbolic-operational ideological alignment, and within-operational ideological alignment. Gray areas are NA values.

We first examine the global distribution of ideological polarization across three manifestations and visualize the results in figure 4. Darker shades represent higher polarization levels. As shown in Panel A, disagreement is most pronounced in South Asia, Africa, and Latin America. In contrast, many advanced economies in North America and Western Europe exhibit moderate levels of disagreement, while the North European societies show the lowest levels observed in our dataset. However, this pattern does not hold for the other two manifestations. Panels B and C reveal that North America, Europe, and Australia have the highest levels of both types of ideological alignment. But societies in Africa, Latin America, and Southeast Asia generally show low scores on these two manifestations. One notable exception is East Asia, where societies do not consistently exhibit high values across three manifestations.

This divergence across manifestations is also evident in table 1, where we list the top ten societies on each manifestation. Almost all societies with the highest levels of disagreement are still in the less developed stage. Yet, the societies ranking highest on symbolic-operational alignment are exclusively advanced democracies. Take the United Kingdom and the United States as examples. The UK ranks fourth and second in symbolic-operational and within-operational ideological alignment, respectively, while the United States ranks sixth and fourth, but neither appears among the top societies for disagreement. These initial observations suggest that two manifestations of ideological alignment may often coevolve, but are not necessarily linked to a similar level of disagreement.

### Negative Correlations Between Disagreement and Ideological Alignment

**Figure 5.**
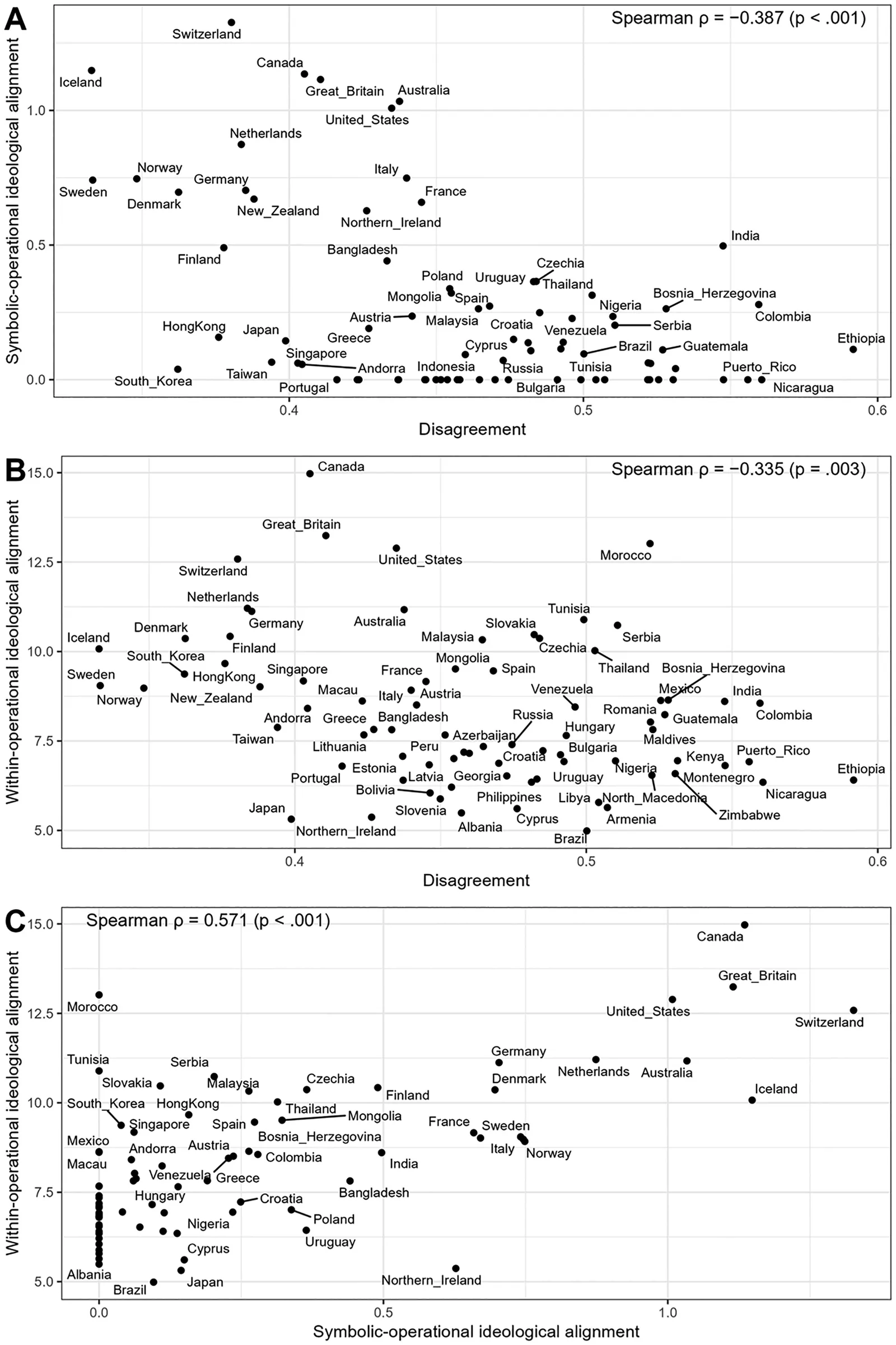
Scatterplots between manifestations of mass ideological polarization. All 78 societies in our sample are displayed as nodes in the figure, while some society labels are omitted in densely overlapping regions to maintain legibility.

We further investigate these relationships by conducting formal statistical tests. Figure 5 presents the pairwise correlation between the three manifestations. First, Panel A suggests that disagreement and symbolic-operational ideological alignment are negatively correlated (Spearman’s ρ = –0.387, *p *< 0.001). This means that ideologically sorted societies may even have lower disagreement on major social issues. In Panel B, a similar negative correlation exists between disagreement and within-operational ideological alignment, though slightly weaker (Spearman’s ρ = –0.335, *p *= 0.003). However, when we move to Panel C, symbolic-operational and within-operational ideological alignment exhibit a high level of positive correlation (Spearman’s ρ = 0.571, *p *< 0.001).

These findings, especially the unexpected negative relationship between ideological divergence and alignment, highlight that mass ideological polarization is more complex than a single construct can capture. While summary measures may remain practical, our results suggest that a dimension-specific approach has the potential for accurately assessing the multifaceted nature of mass ideological polarization across different societies.

### Modernization Predicts Lower Disagreement but Higher Ideological Alignment

**Figure 6.**
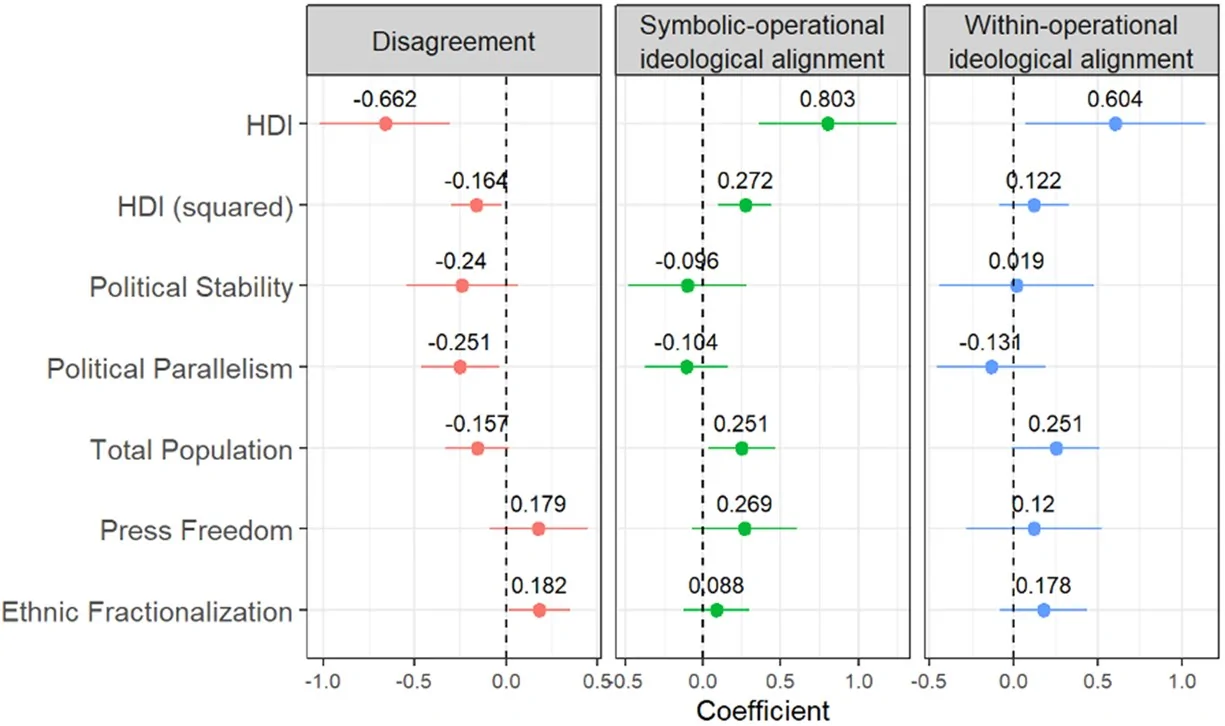
Regression coefficients of OLS models predicting mass ideological polarization. Given the different scales of variables, standardized coefficients are reported, and bars represent 95 percent confidence intervals.

**Figure 7.**
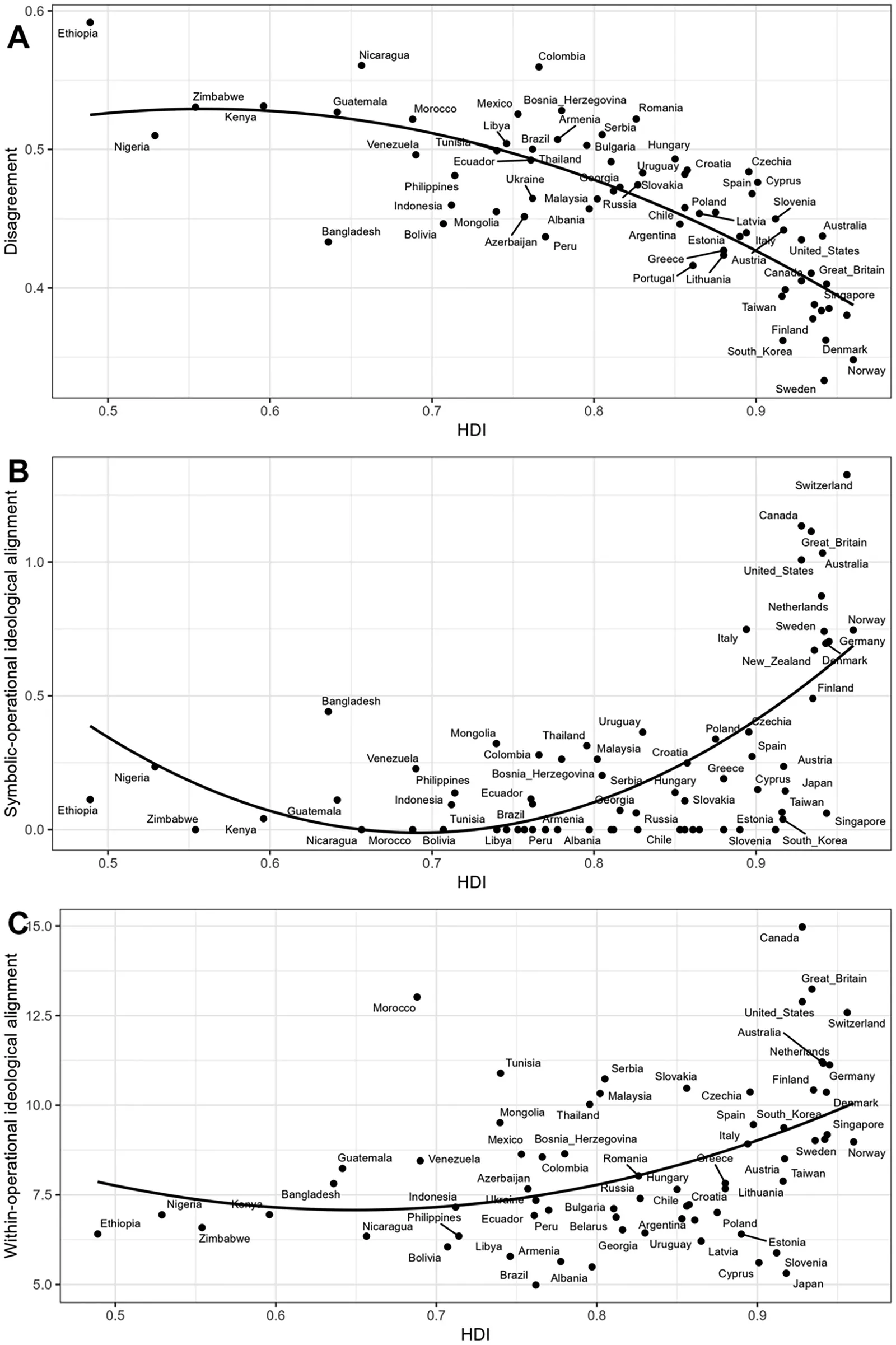
Scatterplots between HDI and each manifestation of mass ideological polarization. The superimposed curves represent fitted quadratic functions.

To examine how modernization predicts ideological polarization, we regressed each polarization manifestation on the HDI, along with several political, social, and institutional variables. Drawing on prior theoretical debates, we included both linear and quadratic terms of HDI to test for potential curvilinear effects. Figure 6 reports the standardized coefficients from OLS regression models. For better interpretation, we also visualize the curvilinear effects on the scatterplots between HDI and each manifestation in figure 7.

As shown in the left panel, HDI is significantly negatively associated with disagreement (β = –0.662, *p *< 0.001), suggesting that opinion dispersion decreases as societies become more modernized. The negative HDI-squared term (β = –0.164, *p *= 0.022) further indicates a nonlinear, accelerating decline. From Panel A in figure 7, we can see that disagreement decreases more steeply at a high development stage.

Yet, the middle panel reveals a strong positive association between HDI and symbolic-operational ideological alignment (β = 0.803, *p *< 0.001), with a significant positive squared term (β = 0.272, *p *= 0.003). In more developed societies, not only are citizens more likely to align their attitudes along ideological lines, but this alignment becomes strengthened as development advances, particularly when HDI exceeds 0.90 (see Panel B in figure 7). For societies with an HDI below 0.90, their symbolic-operational ideological alignment maintains a consistently low level.

A similar pattern emerges for within-operational ideological alignment (the right panel), where HDI also shows a significant positive association (β = 0.604, *p *= 0.031). But its quadratic term is nonsignificant. This indicates a linear trend: Individuals in more developed societies generally exhibit more internally consistent political beliefs.

These patterns also offer a plausible explanation for the negative relationship between disagreement and ideological alignment. Because modernization is negatively associated with disagreement while simultaneously fostering ideological alignment, the observed negative correlations between disagreement and ideological alignment may simply reflect how modernization shapes these manifestations in different pathways. To test this possibility, we estimate additional regression models with disagreement as the dependent variable, ideological alignment as predictors, while controlling for HDI. As shown in table 2, neither symbolic-operational (Model 2) nor within-operational ideological alignment (Model 4) remains significantly correlated with disagreement. Their coefficients also become near zero. Thus, the negative correlation observed earlier may be simply explained by their divergent associations with modernization.

**Table 2.** OLS models predicting disagreement.

	Dependent variable: disagreement			
	(1)	(2)	(3)	(4)
Symbolic-operational ideological alignment	−0.511 (0.107)	−0.070 (0.101)		
Within-operational ideological alignment			−0.319 (0.118)	−0.017 (0.085)
HDI		−0.876 (0.125)		−0.941 (0.106)
HDI-squared		−0.175 (0.071)		−0.203 (0.064)
Adj *R*^2^	0.250	0.610	0.088	0.608
*N*	67	67	67	67

*Note:* Standardized coefficients with standard errors in parentheses.

## Discussion

Recognizing the multidimensional nature of mass ideological polarization, our study is the first to operationalize this understanding cross-contextually by employing a network-based approach. This context-adaptable comparison allows us to empirically clarify the relationships among disagreement, symbolic-operational, and within-operational ideological alignment, which is unattainable in previous single-society analyses.

### Separate Dimensions of Mass Ideological Polarization

Analyzing ideological polarization across three manifestations, we observed distinct patterns across societies. Societies exhibiting low disagreement often demonstrate higher levels of ideological alignment. The correlation test also reveals that disagreement is negatively associated with ideological alignment, suggesting a divergence between these polarization manifestations. This divergence, while unexpected, actually resonates with prior longitudinal evidence suggesting that the rise of partisan sorting does not necessarily track increased disagreement in the United States (Fiorina and Abrams 2008; Jocker et al. 2024) and Canada (Merkley 2023).

One possible macrostructural explanation for this divergence could be modernization, the strongest predictor of all three polarization manifestations in our regression models. After controlling for pertinent factors, we find that modernization negatively predicts disagreement, which is consistent with the Social Transition Model (Bell 1960): Modernization tends to foster social consensus and dampen disagreement on major socioeconomic, survival-related issues. While modernization-driven ideological conflict may apply to a subset of preferences (e.g., identity politics), it may not affect most. On the other hand, the expansion of education, media, and party systems strengthens ideological identities and provides clearer interpretive frameworks for political issues (Keskintürk 2022). Meanwhile, modernization cultivates greater political interest and knowledge among the public (Inglehart and Welzel 2005), enabling citizens to internalize institutionalized elite cues and thereby contributing to a less multidimensional ideological structure. In this sense, modernization channels the uncoordinated opinion diversity into more patterned, institutionalized ideological differentiation. The result is that disagreement becomes less about issue-by-issue division and more about stable ideological boundaries, producing a negative association between overall disagreement and ideological alignment across development levels.

Recognizing the separateness of polarization’s dimensions points to important theoretical implications. Rather than assuming a unified trajectory of growing ideological division, theories of ideological polarization should move beyond general models (Rigoli 2025) and single measures (Mehlhaff 2025) toward frameworks that specify the conditions under which each manifestation emerges and how they interrelate. This coincides with recent efforts to conceptualize affective polarization as a multidimensional construct (Campos and Federico 2026). While summary measures may remain useful for certain comparative or theoretical purposes, we encourage researchers to use them with care in their future work: clearly specifying which manifestation(s) their measures capture and exercising caution when interpreting results as “polarization” in general. Normatively, this perspective also cautions against treating all signs of ideological polarization as inherently harmful. For example, increased within-operational ideological alignment might reflect greater political knowledge (Fishman and Davis 2022) rather than social fragmentation per se; the surging disagreement may arise from group struggling for equality (Kreiss and McGregor 2024).

Notably, both forms of ideological alignment are positively correlated across societies. According to the concept of ideological institutionalization (Grofman 2004), as societies modernize, political parties are able to cultivate clearer ideological profiles, more programmatic competition, and more durable connections with their constituencies (Keskintürk 2022). These features enhance the consistency and visibility of ideological cues in elite and media discourse, also increasing the value of left-right ideological labels (Kozlowski and Murphy 2021). When parties communicate with the public through institutionalized media and education systems, citizens are more likely to interpret issues through shared ideological lenses and align their preferences with ideological identities (Zaller 1992). This institutionalization process strengthens the symbolic boundaries between ideological camps and enhances belief coherence within them, leading to a status in which ideological sorting and issue alignment evolve together.

Besides, the co-occurrence of symbolic-operational and within-operational ideological alignment lends indirect evidence to the idea that treats ideological alignment as an endogenous, mutually reinforcing system. Given the central role of ideological identity as a structuring mechanism in belief systems (Boutyline and Vaisey 2017; Brandt et al. 2019), future research can further examine the potential reciprocal dynamics within ideological polarization. While contexts that strengthen ideological identities tend to produce more coherent belief systems, consistent issue preferences may, in turn, make ideological labels more meaningful and stable.

This study thus underscores the need for an expanded framework to analyze ideological polarization dynamics, incorporating two key axes. The first axis involves differentiating the various manifestations of polarization. Future research, whether single-context or comparative, can benefit from identifying their distinct predictors, configurations, and outcomes and investigating them separately in empirical analyses. The second axis refers to the levels of factors related to ideological polarization, especially in comparative contexts. While previous research has identified within-society factors, such as individual political interest, as predictors of subgroup polarization (Kim 2015; Davis and Dunaway 2016), we argue for the inclusion of between-society factors, such as modernization. The proposed two-axes framework offers a more comprehensive road map for advancing polarization scholarship.

### Belief Network in Comparative Public Opinion Research

Methodologically, this study employs belief network methods to assess ideological alignment. Network measures enable the inclusion of a broad range of political beliefs without imposing a predefined structure, making them particularly useful for comparative studies across varied political cultures. We believe this network approach has significant potential for application in other comparative studies. Previous research on similar networks has explored topics such as the structure of personality domains and behavior co-occurrence (Epskamp and Fried 2018), and the temporal evolution of political belief systems (Fishman and Davis 2022). When combined with comparative research design, this approach can provide insights into the roles of macrostructural factors in various issues.

### Limitations and Future Research

This study has several limitations. First, we do not examine the temporal and group heterogeneity of ideological polarization. Temporal analysis could offer stronger evidence regarding modernization’s role in shaping ideological polarization, and investigating how different social groups exhibit varying polarization patterns, along with the interaction between those within-society and between-society factors, could reveal how structural elements collectively influence mass ideological polarization.

Second, our focus on symbolic-operational ideological alignment differs from the classic literature on partisan sorting, which centers on the alignment between party identification and ideology (Levendusky 2009). We adopt this approach to facilitate cross-national comparability, but this choice means that our measure does not directly capture the extent to which ideological conflict is layered onto partisan competition. Future cross-national research should therefore compare how ideological alignment interacts with partisan identities.

Moreover, many studies have developed strategies to adjust cross-national public opinion panel data, thereby enhancing comparability—particularly for item-level estimates (e.g., Claassen 2019). Yet, the potential extension of these techniques to network models, where relational properties are the central concern, remains underexplored. Advancing such methods would likely improve the comparability of our results and represents an important direction for future research.

Finally, other between-society factors, such as the sorting of social identities (Iyengar et al. 2019), have been extensively discussed. Future studies should explore their roles in explaining polarization dynamics across societies.

## Supplementary Material

nfag037_Supplementary_Data

## Data Availability

Replication data and documentation are available at https://doi.org/10.7910/DVN/Z866VY.

## Appendix A. AAPOR-Required Disclosure Elements

### First Data Source

Cross-national survey data from the European Values Study (EVS) and the World Values Survey (WVS) joint program (2017–2022; version 5-0-0). Secondary data obtained from the EVS archive (GESIS-DAS) and the WVS archive (JDSystems Madrid) via https://www.worldvaluessurvey.org/WVSEVSjoint2017.jsp.

### Data Collection Strategy

Nationally representative surveys of adult populations in participating countries. Each national team conducted face-to-face, telephone, or web-based interviews using standardized questionnaires under EVS/WVS methodological guidelines. See mode of data collection for each survey from the EVS/WVS Joint v5.0 Codebook (pages 8–10) via https://www.worldvaluessurvey.org/WVSEVSjoint2017.jsp.

### Research Sponsor and Conductor

EVS has been responsible for planning and conducting surveys in European countries. The World Values Survey Association (WVSA) has been responsible for planning and conducting surveys in countries in the world outside Europe as well as several European surveys (Andorra, Cyprus, Greece, Northern Ireland). For Principal Investigators in each country, see https://doi.org/10.4232/1.14320.

### Measurement Tools/Instruments

The EVS/WVS Master Questionnaires, translated into national languages according to translation protocols. Instruments included standardized items on moral, religious, political, and social values. Full questionnaires and coding schemes are archived at https://www.worldvaluessurvey.org/WVSEVSjoint2017.jsp.

### Population Under Study

Individuals aged 18 or older residing within private households in each country at the date of beginning of fieldwork (or in the date of the first visit to the household, in case of random-route selection).

### Methods Used to Generate and Recruit the Sample

Nationally representative, single-stage or multistage probability samples. Sampling designs reviewed and approved by the EVS Methodology Group or WVS Scientific Advisory Committee. See details from the EVS/WVS Joint v5.0 Codebook (pages 8–10) via https://www.worldvaluessurvey.org/WVSEVSjoint2017.jsp.

### Methods and Modes of Data Collection

Mixed-mode: face-to-face (CAPI/PAPI), telephone (CATI), and self-administered (CAWI or paper). Mode assignment determined by national design. Conducted in the major languages of each country (EVS: ≥5 percent language threshold; WVS: ≥15 percent). See country-specific details from the EVS/WVS Joint v5.0 Codebook (pages 8–10) via https://www.worldvaluessurvey.org/WVSEVSjoint2017.jsp.

### Dates of Data Collection

January 18, 2017—July 2, 2023 (country-specific fieldwork dates detailed in documentation, see https://doi.org/10.4232/1.14320).

### Sample Sizes and Discussion of the Precision of the Results

156,658 individual respondents across all countries. The sample size was set as effective sample size: a minimum of 1,200 for countries with a population over 2 million and 1,000 for countries with a population below 2 million. Countries with populations over 100 million and greater population diversity are advised to use a sample size of 1,500 respondents and more. The sampling was documented using the Survey Design Form delivered by the national teams, which included the description of the sampling frame and each sampling stage as well as the calculation of the planned gross and net sample size to achieve the required effective sample. See https://www.worldvaluessurvey.org/WVSEVSjoint2017.jsp for more details.

### Whether and How the Data Were Weighted

We report the results from unweighted data in the main text, and provided results using weighted data in Supplementary Material section I. In the EVS/WVS dataset, the weight variable (gwght) is designed to correct for differences in age, sex, educational level, and region of residence between the survey sample and the population benchmark.

### How the Data Were Processed and Procedures to Ensure Data Quality

The Joint EVS/WVS uses international standards and coding frameworks. For EVS, a workflow was implemented with several steps for checking/cleaning and standardization/harmonization of the national data as well as detailed process documentation was agreed. See more details via https://europeanvaluesstudy.eu/methodology-data-documentation/survey-2017/methodology/evs2017-what-has-been-done-to-achieve-good-data-quality/.

### Study Stimuli

Interviewers used standardized show cards corresponding to the EVS/WVS questionnaire.

### Dispositions or Response or Participation Rates

Not reported. Investigators are expected to make every reasonable effort to minimize nonresponse. A full report on nonresponses is required.

### Sample Sizes

Our measures of ideological polarization involve 35 survey items. After excluding all cases with missing data, the final analytic sample includes 74,886 individuals from 78 societies.

### Measurement and Model Specification

See details in the main text.

### General Statement Acknowledging Limitations of the Design and Data Collection

Not applicable for this secondary data analysis.
