# Supplementary material for: Disentangling the Three Facets of Mass Ideological Polarization: A Network Approach Across 78 Societies
Source: Public Opin Q. 2026 Sep 8;90(SI):1017–48. doi: 10.1093/poq/nfag037 (PMC13553011; doi:10.1093/poq/nfag037)
Supplement: Supplementary file 1 [file nfag037_supplementary_data.pdf]

Supplementary Material

Disentangling the Three Facets of Mass Ideological Polarization: A Network Approach Across 78 Societies

Yufan Guo [a], Yilang Peng [b], Tian Yang [a]

[a] School of Journalism and Communication, The Chinese University of Hong Kong, Shatin, Hong Kong SAR.

[b] Department of Financial Planning, Housing and Consumer Economics, University of Georgia, GA, USA

Corresponding author: Tian Yang, Humanities Building, CUHK, Shatin, NT, HKSAR, email: tyang@cuhk.edu.hk

Supplementary Material

Section A: The Full Lists of Selected Items and Societies 3

Section B: Excluding Additional Ideological Items 6

Section C: Country-Level Variables 8

Section D: Country-Year as the Unit of Analysis 10

Section E: Measurement Validation 11

Section F: Measuring Within-operational ideological alignment by Network Density ... 14

Section G: Measuring Modernization by GDP per capita 16

Section H: Belief Network Simulation 18

Section I: Weighted Results 20

Section A: The Full Lists of Selected Items and Societies

Table A1 *The full list of selected items in the WVS/EVS dataset*

	Code	Item [a]	Scale	ρ [b]
1	A124_02	Neighbors: People of a Different Race	0/1	.442
2	A124_06	Neighbors: Immigrants/Foreign Workers	0/1	.361
3	B008	Protecting environment vs. Economic growth	1~3	.410
4	C001_01	Jobs scarce: Men should have more right to a job than women	1~5	.598
5	C002_01	Jobs scarce: Employers should give priority to (nation) people than immigrants	1~5	−.105
6	D059	Men make better political leaders than women do	1~4	.555
7	D060	University is more important for a boy than for a girl	1~4	.569
8	D061	The Children Suffer with Working Mother	1~4	.548
9	D078	Men make better business executives than women do	1~4	.490
10	E035	Income equality	1~10	.729
11	E036	Private vs state ownership of business	1~10	.840
12	E037	Government responsibility	1~10	.829
13	E039	Competition good or harmful	1~10	.772
14	E114	Political System: Strong Leadership	1~4	.484
15	E115	Political System: Experts	1~4	.226
16	E117	Political System: Democratic Politics	1~4	.661
17	E224	Democracy: Governments tax the rich and subsidize the poor.	1~10	.826
18	E225	Democracy: Religious authorities interpret the laws.	1~10	.860
19	E226	Democracy: People choose their leaders in free elections.	1~10	.814
20	E227	Democracy: People receive state aid for unemployment.	1~10	.780
21	E228	Democracy: The army takes over when government is incompetent.	1~10	.779
22	E229	Democracy: Civil rights protect people's liberty against oppression.	1~10	.826
23	E233	Democracy: Women have the same rights as men.	1~10	.796
24	E233A	The State Makes People's Incomes Equal	1~10	.666
25	E233B	People Obey Their Rulers	1~10	.597
26	E235	Importance of Democracy	1~10	.745
27	F118	Justifiable: Homosexuality	1~10	−.027
28	F120	Justifiable: Abortion	1~10	.108
29	F121	Justifiable: Divorce	1~10	.741
30	F123	Justifiable: Suicide	1~10	−.346
31	F144_02	Ethical Values and Norms: Death Penalty	1~10	.510
32	G007_35_B	Trust: People of Another Religion	1~4	.824
33	G007_36_B	Trust: People of Another Nationality	1~4	.799
34	G052	Evaluate the Impact of Immigrants Over Country	1~5	.365
	E033	Self positioning in political scale	1~10	-

Note. [a] For complete statement and scale options, see the codebook from https://www.worldvaluessurvey.org/WVSEVSjoint2017.jsp

[b] We calculated Spearman's ρ correlation between the disagreement of each item and the overall disagreement value, suggesting that the average disagreement value is not driven by just a few items but reflects a general trend.

Table A2 *The full list of selected societies in the WVS/EVS dataset*

	Country/Region	Source	Sample Size	Year
1	Albania	EVS	1435	2018
2	Andorra	WVS	1004	2018
3	Argentina	WVS	1003	2017
4	Armenia	EVS/WVS	2723	2018; 2021
5	Australia	WVS	1813	2018
6	Austria	EVS	1644	2018
7	Azerbaijan	EVS	1800	2018
8	Bangladesh	WVS	1200	2018
9	Belarus	EVS	1548	2018
10	Bolivia	WVS	2067	2017
11	Bosnia and Herzegovina	EVS	1724	2019
12	Brazil	WVS	1762	2018
13	Bulgaria	EVS	1558	2017; 2018
14	Canada	WVS	4018	2020
15	Chile	WVS	1000	2018
16	Colombia	WVS	1520	2018
17	Croatia	EVS	1487	2017; 2018
18	Cyprus	WVS	1000	2019
19	Czechia	EVS/WVS	3011	2017; 2022
20	Denmark	EVS	3362	2017; 2018
21	Ecuador	WVS	1200	2018
22	Estonia	EVS	1304	2018
23	Ethiopia	WVS	1230	2020
24	Finland	EVS	1199	2017; 2018
25	France	EVS	1870	2018
26	Georgia	EVS	2194	2018
27	Germany	EVS/WVS	3698	2017; 2018
28	Great Britain	EVS/WVS	4397	2018; 2022
29	Greece	WVS	1200	2017
30	Guatemala	WVS	1203	2019; 2020
31	Hong Kong SAR	WVS	2075	2018
32	Hungary	EVS	1514	2018
33	Iceland	EVS	1624	2017; 2018
34	India	WVS	1692	2023
35	Indonesia	WVS	3200	2018
36	Italy	EVS	2277	2018; 2019
37	Japan	WVS	1353	2019
38	Kenya	WVS	1266	2021
39	Latvia	EVS	1335	2021
40	Libya	WVS	1196	2021; 2022
41	Lithuania	EVS	1448	2017; 2018
42	Macau SAR	WVS	1023	2019

43	Malaysia	WVS	1313	2018
44	Maldives	WVS	1038	2021
45	Mexico	WVS	1739	2018
46	Mongolia	WVS	1638	2019; 2020; 2021
47	Montenegro	EVS	1003	2019
48	Morocco	WVS	1200	2021
49	Netherlands	EVS/WVS	4549	2017; 2018; 2022
50	New Zealand	WVS	1057	2019; 2020
51	Nicaragua	WVS	1200	2019; 2020
52	Nigeria	WVS	1237	2017; 2018
53	North Macedonia	EVS	1117	2018; 2019
54	Northern Ireland	WVS	447	2022
55	Norway	EVS	1122	2018
56	Peru	WVS	1400	2018
57	Philippines	WVS	1200	2019
58	Poland	EVS	1352	2017; 2018
59	Portugal	EVS	1215	2020
60	Puerto Rico	WVS	1127	2018
61	Romania	EVS/WVS	2870	2017; 2018
62	Russia	EVS/WVS	3635	2017
63	Serbia	EVS/WVS	2545	2017; 2018
64	Singapore	WVS	2012	2019; 2020
65	Slovakia	EVS/WVS	2632	2017; 2022
66	Slovenia	EVS	1075	2017
67	South Korea	WVS	1245	2017; 2018
68	Spain	EVS	1209	2017; 2018
69	Sweden	EVS	1194	2017; 2018
70	Switzerland	EVS	3174	2017; 2018
71	Taiwan ROC	WVS	1223	2019
72	Thailand	WVS	1500	2017; 2018
73	Tunisia	WVS	1208	2019
74	Ukraine	EVS/WVS	2901	2020
75	United States	WVS	2596	2017
76	Uruguay	WVS	1000	2022
77	Venezuela	WVS	1190	2021
78	Zimbabwe	WVS	1215	2020

Note. While the EVS/WVS project surveyed 92 societies, it did not measure self-placed ideology in 12 societies: China, Egypt, Iran, Iraq, Jordan, Kazakhstan, Kyrgyzstan, Lebanon, Myanmar, Pakistan, Uzbekistan, Vietnam. Besides, another issue item was not measured in Tajikistan and Turkey, so our final sample include respondents from 78 societies.

Section B: Excluding Additional Ideological Items

Our ideology-related items include three issue domains. *Economic* and *social* dimensions are two major dimensions in the traditional framework of operational ideology. (Jost et al., 2009). We added a third dimension, *governmental/democratic*, to capture preferences for different types of government and understandings of democracy in other non-Western societies. While in many Western democracies, support for liberal democracy over alternative regime types is largely taken for granted, this consensus does not necessarily hold in other societies, such as China (Pan & Xu, 2018) and Latin America (Zechmeister & Corral, 2013). Table B1 lists the category of each item we specified:

Table B1 *The full list of selected items in the WVS/EVS dataset*

Code	Item	Category
A124_02	Neighbors: People of a Different Race	Social
A124_06	Neighbors: Immigrants/Foreign Workers	Social
B008	Protecting environment vs. Economic growth	Economic
C001_01	Jobs scarce: Men should have more right to a job than women	Social
C002_01	Jobs scarce: Employers should give priority to (nation) people than immigrants	Social
D059	Men make better political leaders than women do	Social
D060	University is more important for a boy than for a girl	Social
D061	The Children Suffer with Working Mother	Social
D078	Men make better business executives than women do	Social
E035	Income equality	Economic
E036	Private vs state ownership of business	Economic
E037	Government responsibility	Economic
E039	Competition good or harmful	Economic
E114	Political System: Strong Leadership	Governmental
E115	Political System: Experts	Governmental
E117	Political System: Democratic Politics	Governmental
E224	Democracy: Governments tax the rich and subsidize the poor.	Governmental/Economic
E225	Democracy: Religious authorities interpret the laws.	Governmental
E226	Democracy: People choose their leaders in free elections.	Governmental
E227	Democracy: People receive state aid for unemployment.	Governmental/Economic
E228	Democracy: The army takes over when government is incompetent.	Governmental
E229	Democracy: Civil rights protect people's liberty against oppression.	Governmental
E233	Democracy: Women have the same rights as men.	Governmental/Social
E233A	The State Makes People's Incomes Equal	Economic
E233B	People Obey Their Rulers	Social
E235	Importance of Democracy	Governmental
F118	Justifiable: Homosexuality	Social
F120	Justifiable: Abortion	Social
F121	Justifiable: Divorce	Social
F123	Justifiable: Suicide	Social
F144_02	Ethical Values and Norms: Death Penalty	Social

G007_35_B	Trust: People of Another Religion	Social
G007_36_B	Trust: People of Another Nationality	Social
G052	Evaluate the Impact of Immigrants Over Country	Social

We replicated the analyses on only social and economic items. For inter-manifestation relationships, disagreement is negatively correlated with symbolic-operational ideological alignment (Spearman's $\rho = -.36$, $p = .001$) and within-operational ideological alignment (Spearman's $\rho = -.39$, $p < .001$). Symbolic-operational ideological alignment and within-operational ideological alignment are positively correlated (Spearman's $\rho = .64$, $p < .001$). For regression results, Figure B1 shows consistent patterns with our findings in the main text. Thus, our findings are not driven by the amalgamation of different item categories.

Figure B1 *Regression coefficients of OLS models predicting mass ideological polarization*

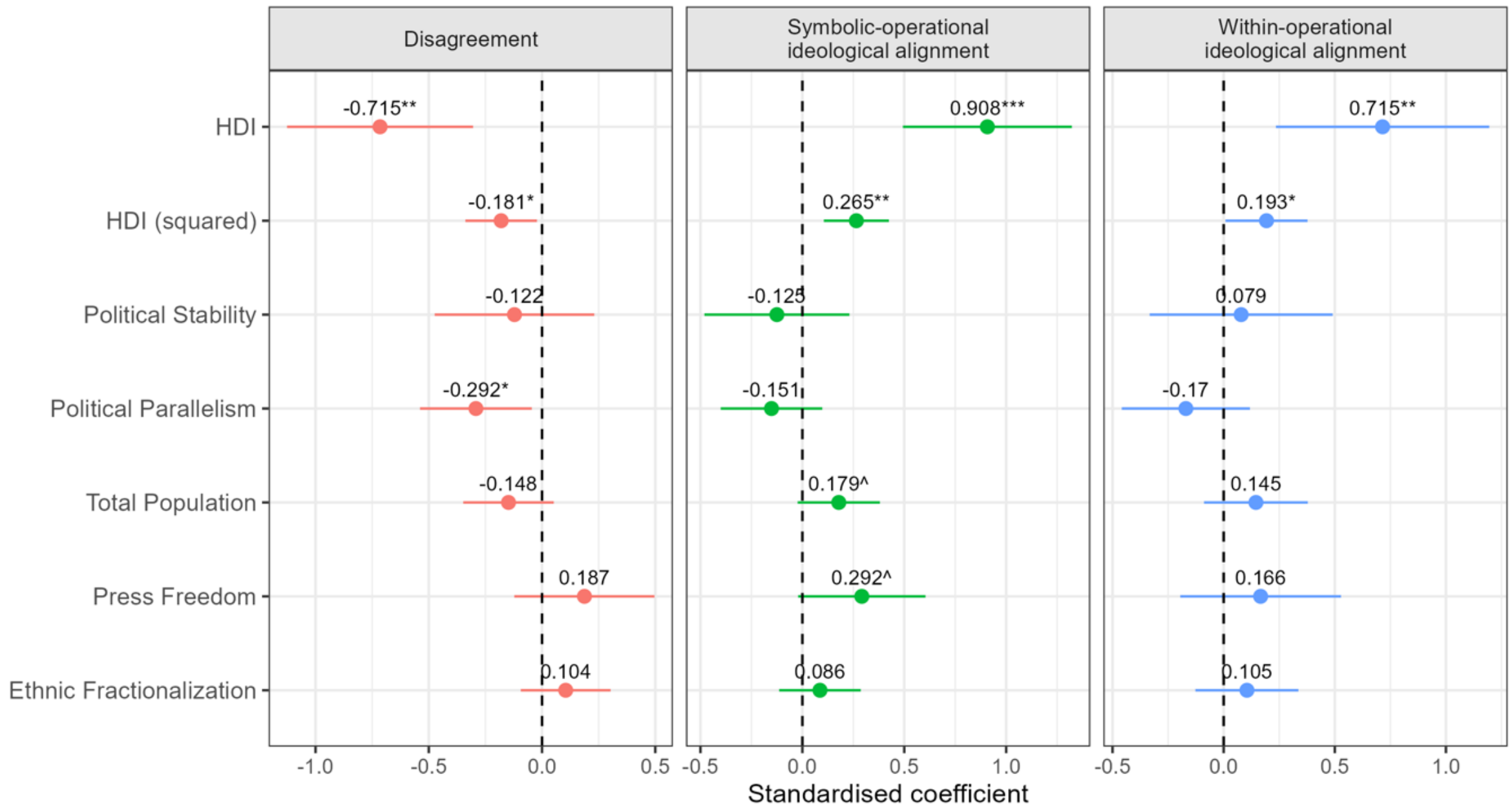

Note. Standardized coefficients are reported, and bars represent 95% confidence intervals.
$^{*}p < .05$, $^{**}p < .01$, $^{***}p < .001$.

Section C: Country-Level Variables

This study relies on multiple data sources to test the relationships between modernization and mass ideological polarization. We provide each country-level variable's source and brief illustration in Table C1, and their descriptive information before the rescaling exercise in Table C2. Here are some additional details.

UNDP is a widely recognized and authoritative source for human development metrics. The Human Development Index (HDI) is a composite measure to assess average achievement in three fundamental dimensions of human development: health (life expectancy), education (mean and expected years of schooling), and standard of living (gross national income per capita). The index ranges from 0 to 1, with higher values indicating greater human development. See https://hdr.undp.org/data-center/human-development-index#/indicies/HDI.

World Bank national accounts data provide the GDP per capita (current US$) for each country, see https://data.worldbank.org/indicator/NY.GDP.PCAP.CD.

The Worldwide Governance Indicators (WGI) published by the World Bank (https://www.worldbank.org/en/publication/worldwide-governance-indicators) compiles data from multiple expert-based and survey-based sources to aggregate indicators for over 200 economies over the period 1996–2023. As one of the six dimensions of governance, *Political Stability and Absence of Violence/Terrorism* measures perceptions of the likelihood of political instability and/or politically motivated violence, including terrorism. The index ranges from −2.5 to 2.5, with higher values indicating greater political stability.

WGI also provides the total national population data (https://databank.worldbank.org/reports.aspx?source=2&series=SP.POP.TOTL&country=#). We log-transformed it given its high skewness.

Press freedom measures the degree to which media can operate independently without government control or censorship. This index is provided by Reporters Without Borders (https://rsf.org/en/index), an internationally watchdog organization that annually publishes the Press Freedom Index, ranging from 0 to 100 (with higher scores indicating more freedom).

Political parallelism refers to the degree to which media content aligns with political party positions or reflects partisan divisions in society. This measure is taken from the *Media Bias* (v2mebias) item from Varieties of Democracy (V-Dem) project, which asked the country experts to rate "Is there media bias against opposition parties or candidates?" (Coppedge et al., 2024). The index ranges from −5 to 5, with higher values denoting greater alignment between media and political actors.

Table C1 *Country-level variables used in this study*

Index	Illustration	Range	Source
HDI	A summary measure of average achievement in key dimensions of human development	0 ~ 1	United Nations Development Programme
GDP per capital	Gross Domestic Product divided by midyear population (Log-transformed)	-	World Bank

Political stability	Perceptions of the likelihood of political instability and/or politically-motivated violence	−2.5 ~ 2.5	Worldwide Governance Indicators, World Bank
Political parallelism	The extent to which media or political parties reflect certain similar views	−5 ~ 5	V-Dem
Total population	(Log-transformed)	-	Worldwide Governance Indicators, World Bank
Press freedom	The degree of independence of the media to operate without government interference	0 ~ 100	Reporters without borders
Ethnic fractionalization	The probability that two randomly drawn individuals are not from the same ethnicity	0 ~ 1	Drazanova (2019)

Table C2 *Descriptive statistics (before rescaled)*

Variable	*Mean*	*SD*	*Min*	*Max*	*N*
HDI	0.82	0.11	0.49	0.96	67
GDP per capital	9.41	1.14	6.81	11.34	67
Political stability	0.08	0.86	−2.25	1.46	67
Political parallelism	1.31	0.92	−1.41	3.06	67
Total population	16.67	1.40	14.07	19.60	67
Press freedom	70.78	12.67	40.27	92.37	67
Ethnic fractionalization	0.37	0.22	0.02	0.86	67
Symbolic-operational ideological alignment	0.26	0.34	0	1.33	67
Within-operational ideological alignment	8.33	2.17	0.04	0.23	67
Disagreement	0.46	0.05	0.33	0.59	67

Note. The final sample consists of 67 societies due to missing data on some variables.

Section D: Country-Year as the Unit of Analysis

In some countries, the WVS survey was fielded in different years. In our main text, we aggregated sample from different survey year at the country level. Here, regarding temporal differences in our data, we conducted an additional robustness check by treating *country-year* as the unit of analysis, thereby separating respondents from the same country who were surveyed in different years. This step increased the number of observations in our regression analyses from 67 to 96. As Figure D1 shows, the regression results remain largely consistent with our main analyses. This suggests that, despite variations in the timing of data collection, the patterns of mass ideological polarization we observe are robust across different years within the same country.

Figure D1 *Regression coefficients of OLS models predicting mass ideological polarization (country-year as the unit of analysis, N = 96)*

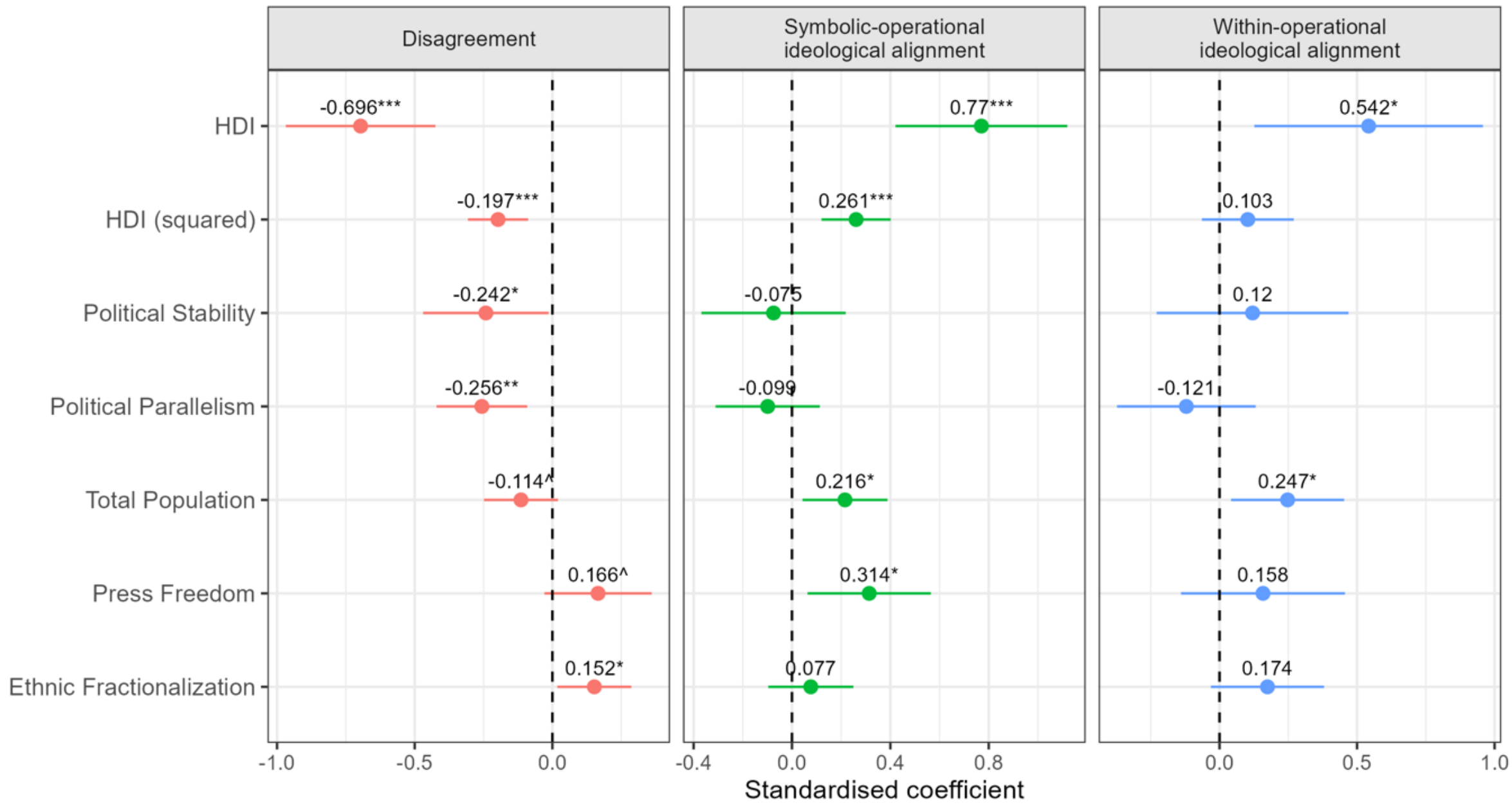

Note. Standardized coefficients are reported, and bars represent 95% confidence intervals. $^{*}p < .05$, $^{**}p < .01$, $^{***}p < .001$.

Section E: Measurement Validation

Using Alternative Measures

To validate our own measures, we first compared them with several existing popular measures mentioned in Table 1, using the following:

- *Disagreement:* ordinal dispersion (ours); bimodality coefficient; averaged variance.
- *Symbolic-operational ideological alignment:* indegree centrality (ours); pairwise correlations
- *Within-operational ideological alignment:* sum of edge weights (ours); network density; pairwise correlations.

Figure E1 shows the Spearman correlation coefficients among these measures. Blue and red numbers denote positive and negative correlations, respectively.

First, there are blue numbers clustering within each manifestation' measures in general, suggesting disagreement (i.e., ideological divergence) and symbolic-operational ideological alignment/within-operational ideological alignment (i.e., ideological consistency) are indeed separate manifestations. One exception is "Within-operational ideological alignment (pairwise correlations)": it is more correlated with symbolic-operational ideological alignment measures, rather than other within-operational ideological alignment measures. But given their shared conceptual foundations, Figure E1 still demonstrates relatively high construct validity.

Second, there is a consistent pattern between disagreement's measures and other two manifestations. All of their correlations are negative (red numbers), suggesting that our findings on the inter-manifestation correlation are not just an artifact of measurement. Rather, most existing measures will generate similar results in our analytical framework.

Figure E1 *Correlation matrix among multiple measures of mass ideological polarization*

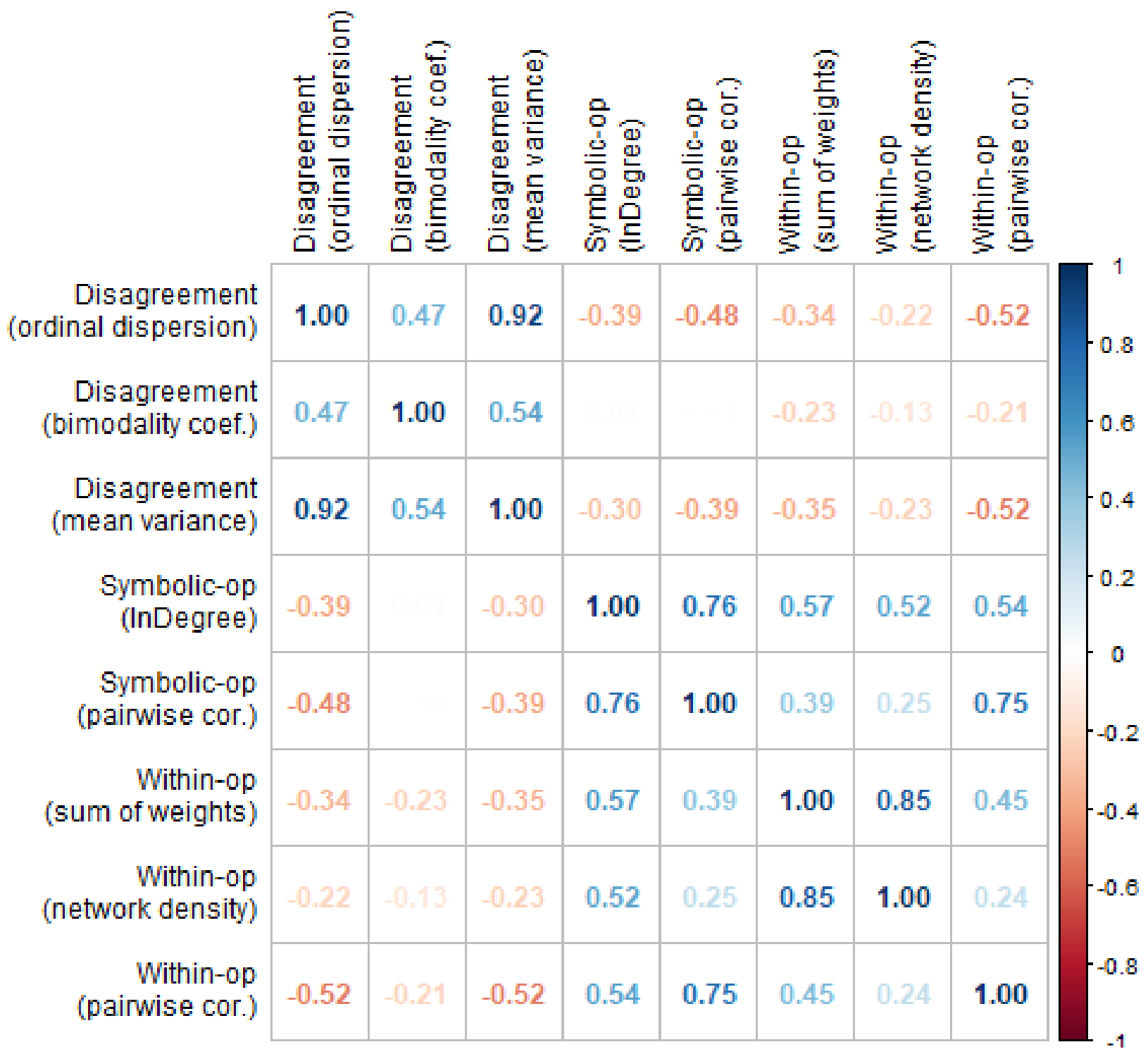

Using Theoretically Related Variables

We also validated our measures by testing the correlation with three indicators that should theoretically be correlated with. Since most previous research on ideological polarization relies on aggregate indicators, there is limited prior literature that provides clear national-level predictors for each specific manifestation we analyze. Thus, we went through related indicators from V-Dem, which currently provides one of the most comprehensive sets of cross-national political variables. Our goal in the validation is therefore to examine whether variables that are conceptually related to a particular manifestation of ideological polarization.

Specifically, for disagreement, we expect it to be related to the overall intensity of societal political divisions. Disagreement captures the extent to which opinions are dispersed across the public, which should be greater in societies where political conflict is more pronounced. We therefore select indicators that capture different forms of societal cleavage or antagonism:

- Political stability (Worldwide Governance Indicators, World Bank): This indicator measures the extent to which a country experiences political unrest, violence, or major institutional disruption (see Table A3). Lower political stability typically reflects deeper political conflict and societal fragmentation, which should correspond to greater disagreement in political attitudes.
- Issue polarization (V-Dem: v2smpolsoc): This reversed-coded measure directly captures the extent to which society is divided over major political issues. 4 means there are differences in opinions but there is a general agreement on the direction for key political issues, and 0 means that there are serious differences in opinions in society on almost all key political issues, which result in major clashes of views. Higher levels of issue polarization here thus indicate less disagreement over policy directions, which should translate into greater disagreement in mass opinion distributions.

For symbolic-operational ideological alignment, we argue that the institutionalization and structure of party competition should be an important predictor. When party systems are institutionalized and programmatic, political actors have stronger incentives to organize political discourse around coherent ideological positions. This, in turn, provides clearer cues for citizens to align their ideological identities with issue attitudes. We therefore include several V-Dem indicators capturing the institutionalization or programmatic clarity of party competition:

- Party system institutionalization (V-Dem: v2xps_party): This variable captures the extent to which parties have stable organizational structures, clear identities, and enduring links to voters. More institutionalized party systems tend to structure political competition along consistent ideological lines, which should encourage stronger alignment between ideological identification and issue positions.
- Party competition across regions (V-Dem: v2pscomprg): This indicator measures whether major parties compete broadly across the national territory or primarily within localized regions. 2 means that most major parties are competitive in most

regions of the country, and 0 means that most major parties are competitive in only one or two regions of the country. When parties compete nationally rather than regionally, political conflict is more likely to be organized around ideological programs rather than local or clientelistic concerns, which should strengthen ideological alignment.

- Distinct party platforms (V-Dem: v2psplats): This variable captures how many political parties with representation in the national legislature or presidency have publicly available party platforms (manifestos) that are publicized and relatively distinct from one another. 4 means all. When parties present clear and differentiated policy agendas, citizens receive stronger informational cues linking ideological identities to specific issue positions, thereby promoting symbolic-operational alignment.

As shown in the Figure E2, the empirical patterns are broadly consistent with these expectations. Indicators capturing societal political divisions, such as issue polarization and political stability, are most strongly associated with our measure of disagreement. In contrast, indicators reflecting the institutionalization and programmatic structure of party competition, including party system institutionalization, nationalized party competition, and distinct party platforms, show associations with symbolic-operational ideological alignment. These patterns provide convergent evidence that our proposed measures capture distinct manifestations of ideological polarization.

Figure E2 *Correlations between mass ideological polarization and five indicators*

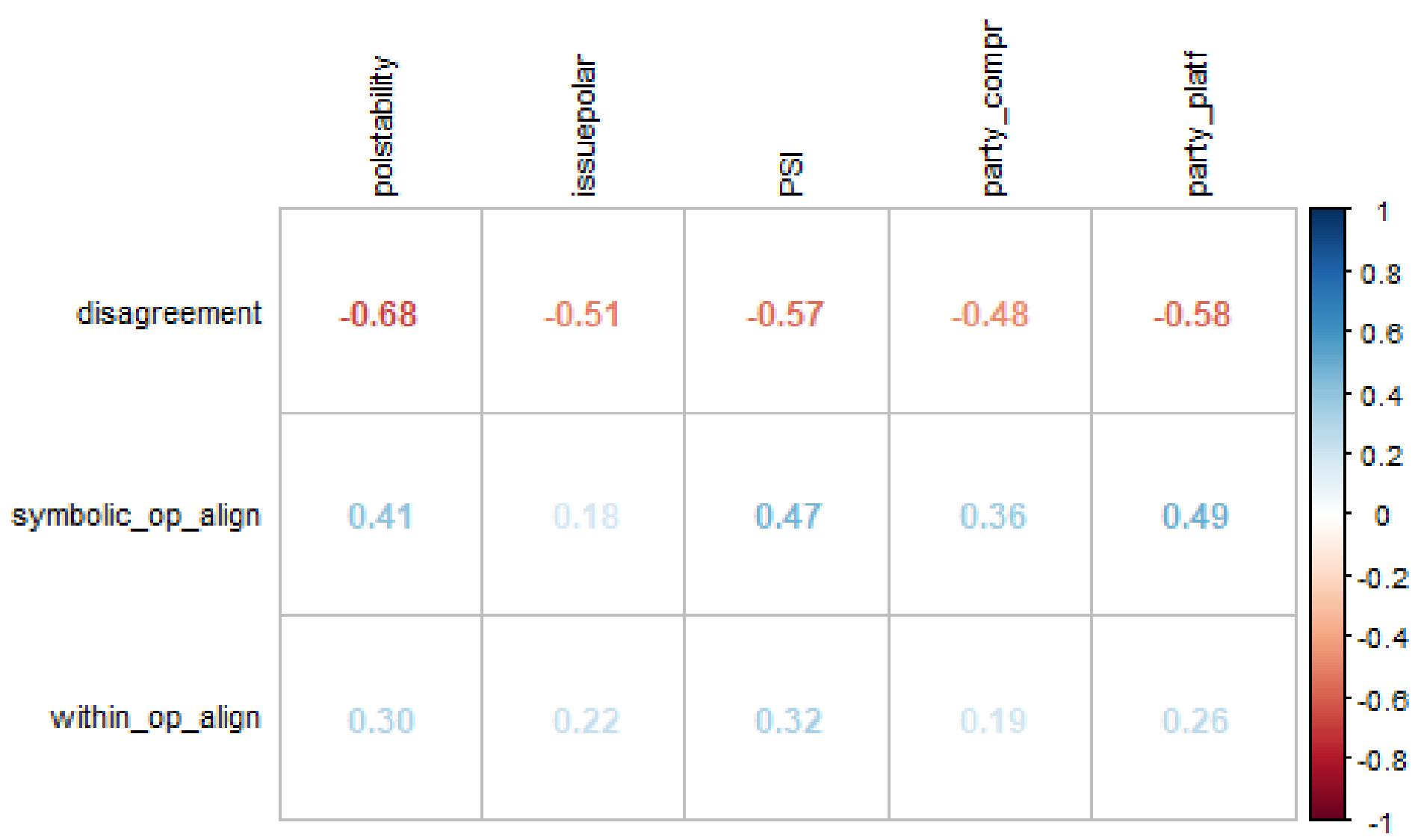

Note. Issue polarization is reversed-coded, so a higher value means less polarized.

Section F: Measuring Within-Operational Ideological Alignment by Network Density

In the main text, we operationalized within-operational ideological alignment using the sum of edge weights. We introduce an alternative measure: network density, a standard measure in network analysis that captures the proportion of existing ties relative to all possible ties in an unweighted network. We replicate our key analyses using this alternative measure to ensure robustness.

First, we assessed the relationship between network density and the sum of edge weights, which is highly correlated (Spearman's $\rho = 0.85$, $p < .001$). Besides, as shown in Figure F1, network density is negatively correlated with disagreement (Spearman's $\rho = –.216$, $p = .057$), but positively with symbolic-operational ideological alignment (Spearman's $\rho = .524$, $p < .001$). These patterns align with those observed for network density, though the magnitude of correlation is slightly stronger for the sum of edge weights.

Second, we visualized the geographical distribution of this measure in Figure F2, which is also highly similar the pattern measured by network density.

Third, we conducted a replication of our regression analysis using the sum of edge weights as the dependent variable while maintaining the same predictor variables. As shown in Figure F3, HDI is positively related to sum of weights ($\beta = 0.563$, $p = .048$), but the squared term is not significant. Therefore, the within-operational ideological alignment-modernization relationship is robust to different network specifications.

Figure F1 *Correlation matrix among manifestations of mass ideological polarization*

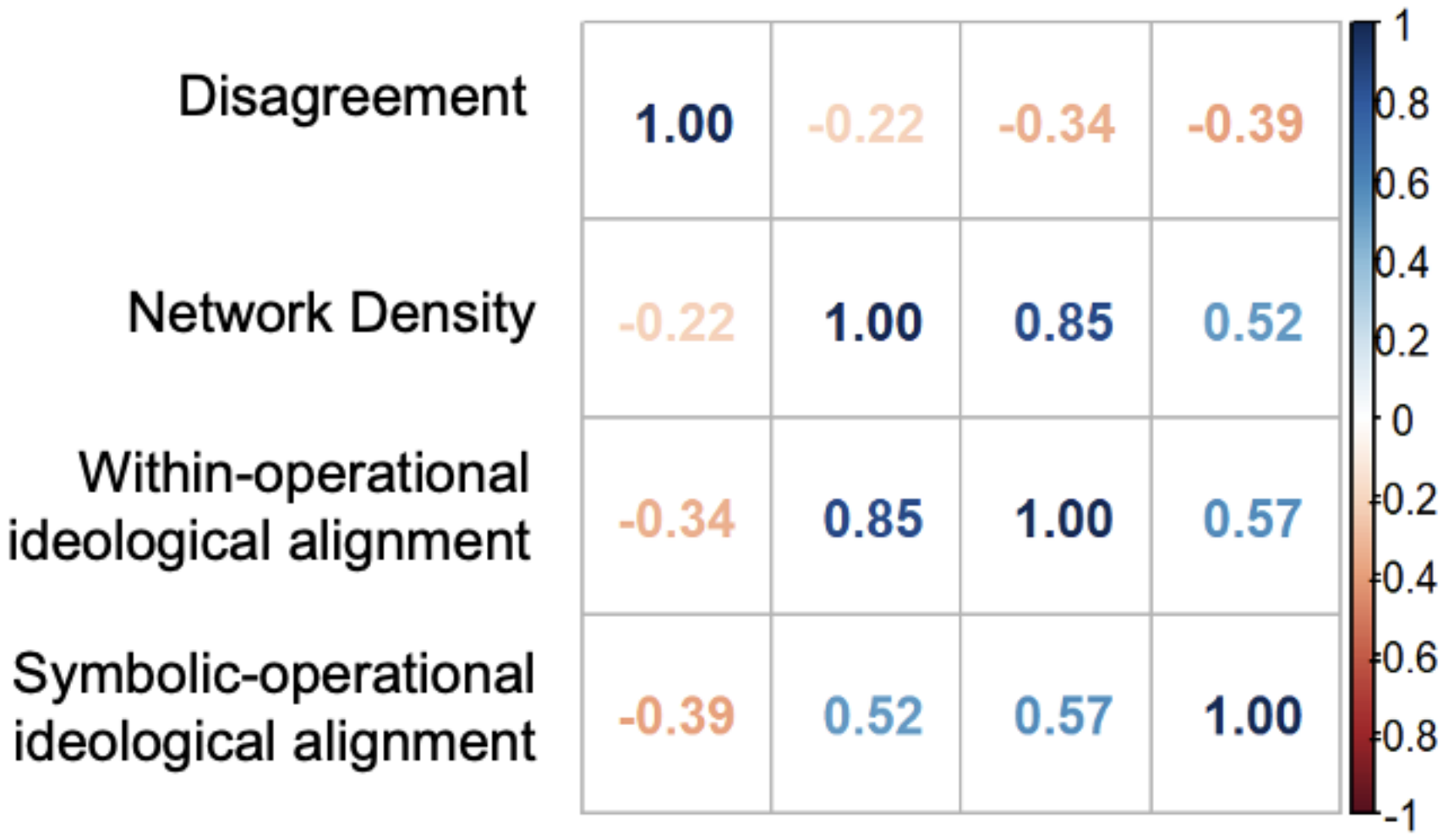

Figure F2 *The global distribution of within-operational ideological alignment (measured by network density)*

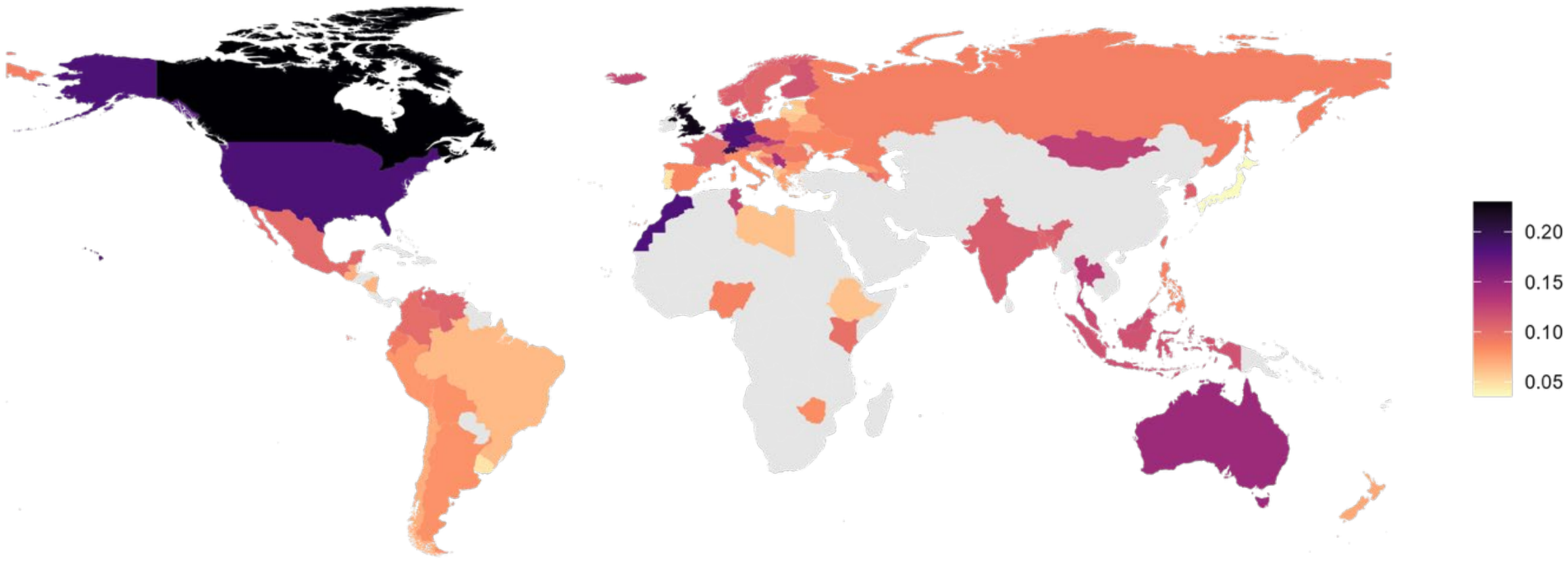

Note. Grey areas are NA values.

Figure F3 *Regression coefficients of OLS models predicting within-operational ideological alignment measured by network density*

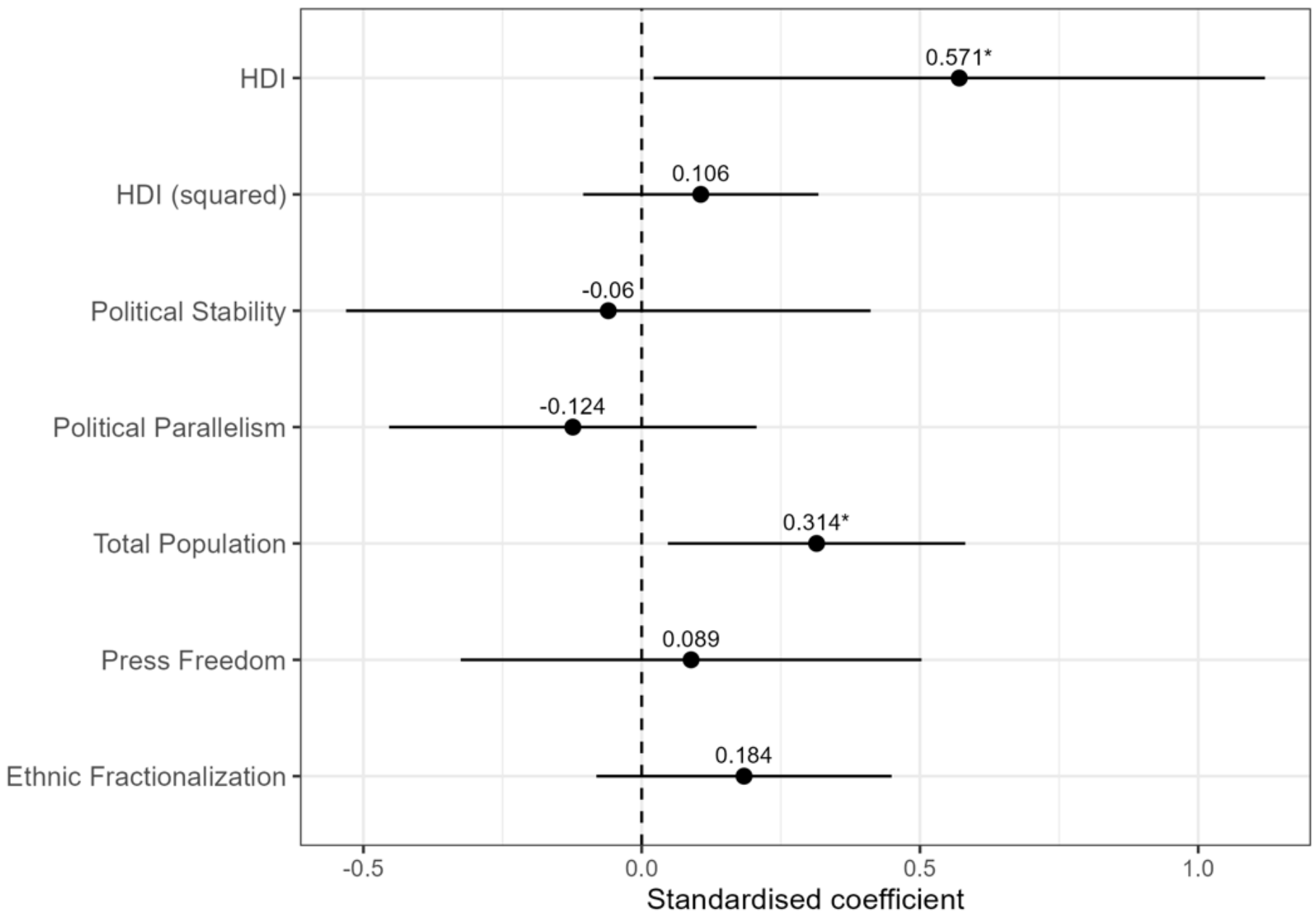

Note. Standardized coefficients are reported, and bars represent 95% confidence intervals.
$^{*}p < .05$.

Section G: Measuring Modernization by GDP per capita

In the main text, we operationalized modernization using the HDI index. Here, we used another common proxy for modernization, GDP per capita, as a robustness check.

Our replication of the regression analysis (Figure G1) reveals generally consistent results. GDP per capita and its squared term are negatively associated with disagreement, and positively associated with symbolic-operational ideological alignment. Its relationship with within-operational ideological alignment, while positive, is only marginally significant.

The scatter plots in Figure G2 further substantiate these patterns, demonstrating consistency with our original HDI-based visualizations in Figure 6.

These results strengthen our confidence in the argument that modernization, whether measured comprehensively through HDI or economically through GDP, predicts lower disagreement and higher symbolic-operational ideological alignment.

Figure G1 *Regression coefficients of OLS models predicting mass ideological polarization*

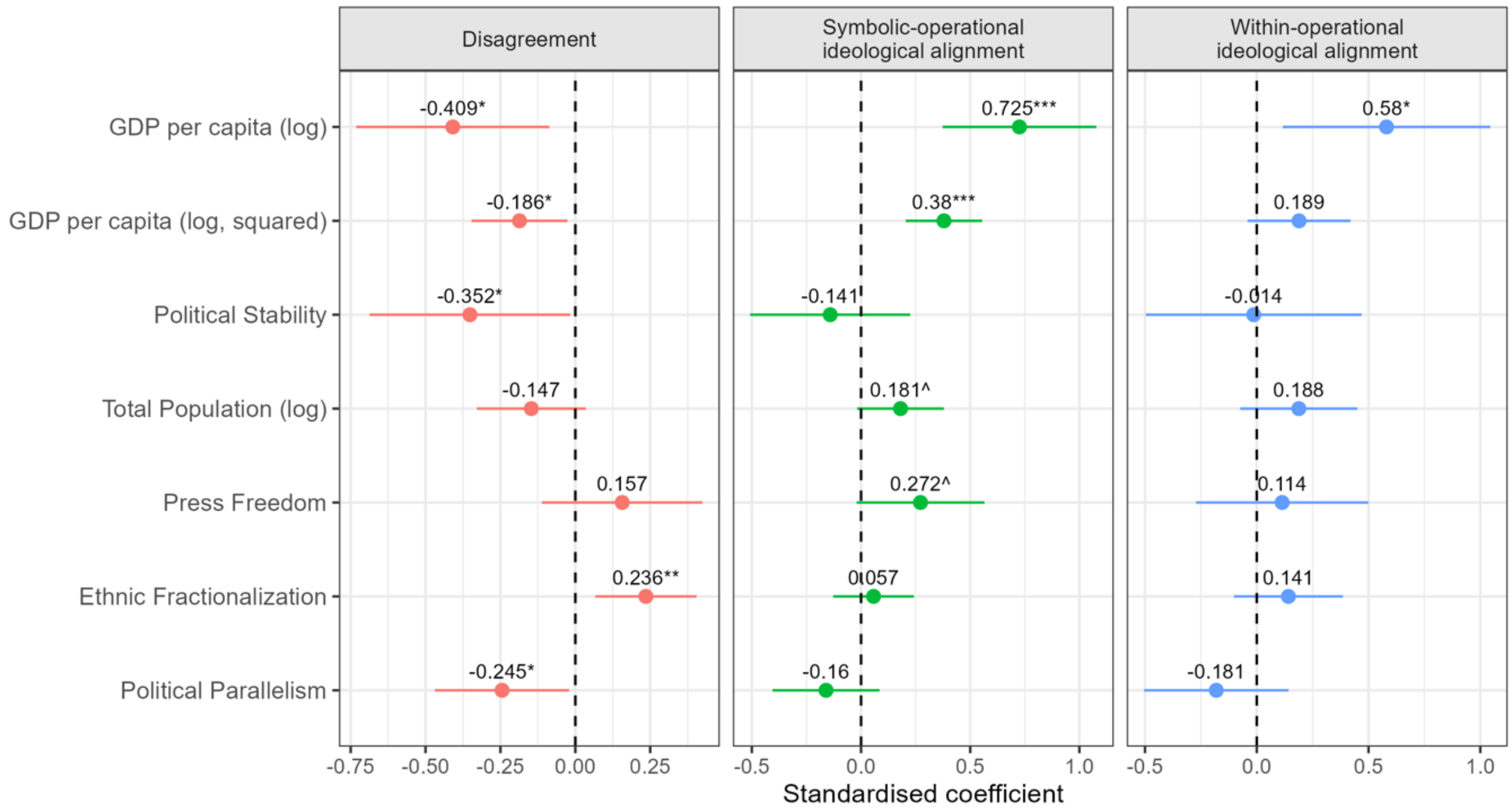

Note. Standardized coefficients are reported, and bars represent 95% confidence intervals. $^{*}p < .05$, $^{**}p < .01$, $^{***}p < .001$.

Figure G2 *Scatter plots between GDP per capita and each manifestation of polarization*

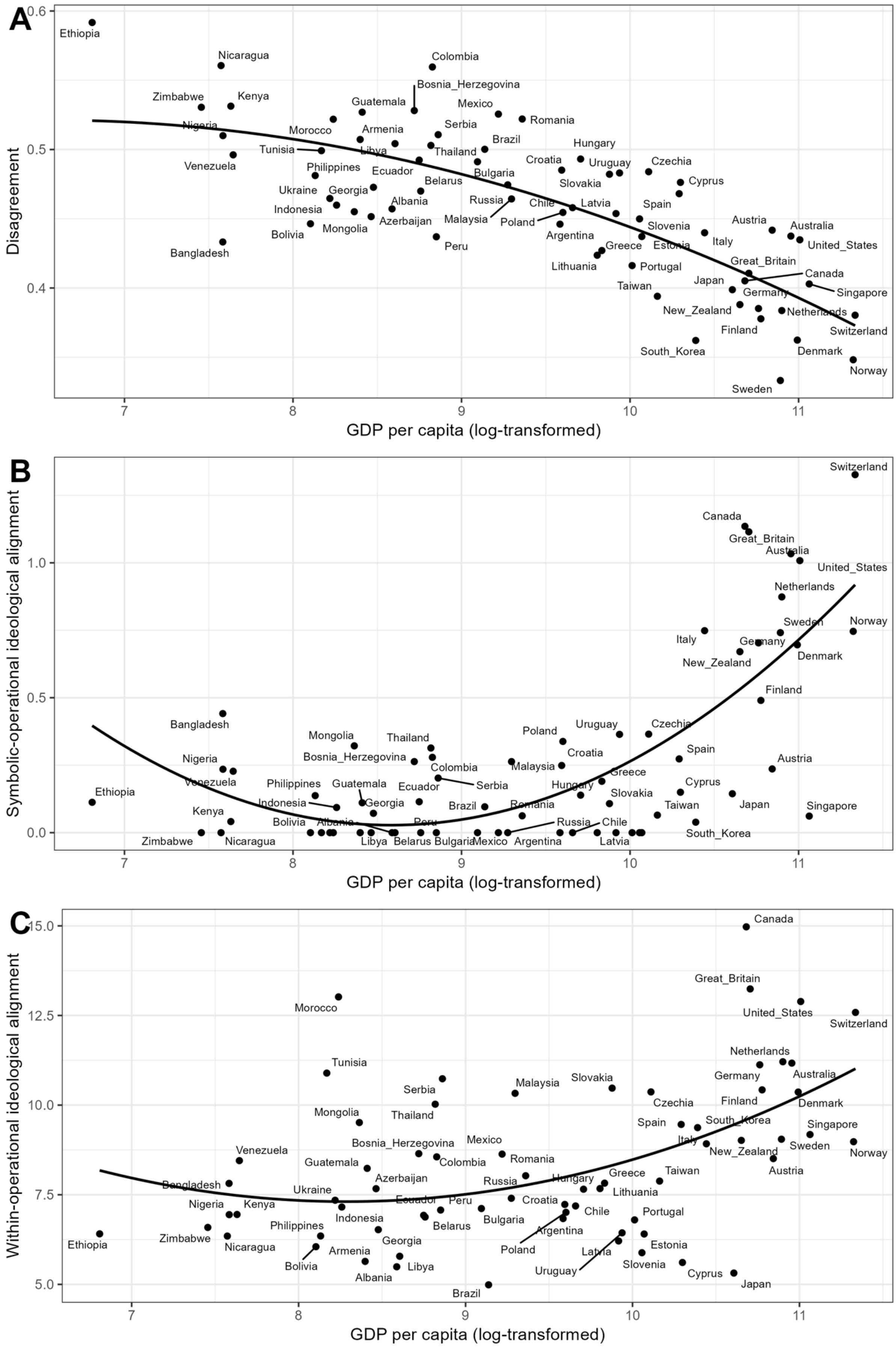

Section H: Belief Network Simulation

In this part, we detail our simulation to illustrate how naïve correlation can overestimate the tie strength between two items.

We generated data for 1,000 simulated individuals on eight attitudinal variables (A–H) under a pre-specified model, where only adjacent items were conditionally dependent (e.g., A–B, B–C, C–D, and so forth) after controlling for all other variables. To characterize conditional dependencies, we began by specifying a precision matrix, which is the inverse of the covariance matrix. If entry $i, j = 0$ in the precision matrix, this means that variables i and j are conditionally independent. In contrast, a nonzero entry indicates that variables i and j still share a "direct connection" that cannot be explained away by the influence of other variables. In our simulation, diagonal elements equaled 1, off-diagonal elements between adjacent nodes were set to 0.3, and no direct connections existed between non-adjacent nodes. The inverse matrix in our simulation can be written as follows.

$$\begin{bmatrix} 1 & 0.3 & 0 & 0 & 0 & 0 & 0 & 0 \\ 0.3 & 1 & 0.3 & 0 & 0 & 0 & 0 & 0 \\ 0 & 0.3 & 1 & 0.3 & 0 & 0 & 0 & 0 \\ 0 & 0 & 0.3 & 1 & 0.3 & 0 & 0 & 0 \\ 0 & 0 & 0 & 0.3 & 1 & 0.3 & 0 & 0 \\ 0 & 0 & 0 & 0 & 0.3 & 1 & 0.3 & 0 \\ 0 & 0 & 0 & 0 & 0 & 0.3 & 1 & 0.3 \\ 0 & 0 & 0 & 0 & 0 & 0 & 0.3 & 1 \end{bmatrix}$$

By inverting this precision matrix, we obtained the covariance matrix used to generate 1,000 simulated observations. Next, we constructed two different types of correlation matrices and visualized them as networks in Figure H1.

First, we computed the regular pairwise correlation matrix. We then plotted this matrix as a network using the *qgraph* package, where each edge reflects the strength of correlation between two variables. In Panel A, many edges appear between non-adjacent nodes (e.g., A–C, E–G), even though there are no direct connections between them. These extra edges reflect spurious, indirect associations mediated through intermediate variables.

Second, we estimated a sparse partial correlation matrix using the graphical lasso (*glasso*). This method applies a regularization procedure that sets small or spurious correlations to zero, leaving only the strongest conditional dependencies. In other words, edges in this network represent direct links between variables after controlling for all others. We again used *qgraph* to visualize the resulting structure, which is much sparser and closer to the precision matrix we originally specified in the simulation (Panel B).

This comparison demonstrates why using simple correlations can overestimate the density and strength of connections, potentially exaggerating the coherence of opinion structures.

Figure H1 *Correlation-based vs. partial correlation network from simulated data*

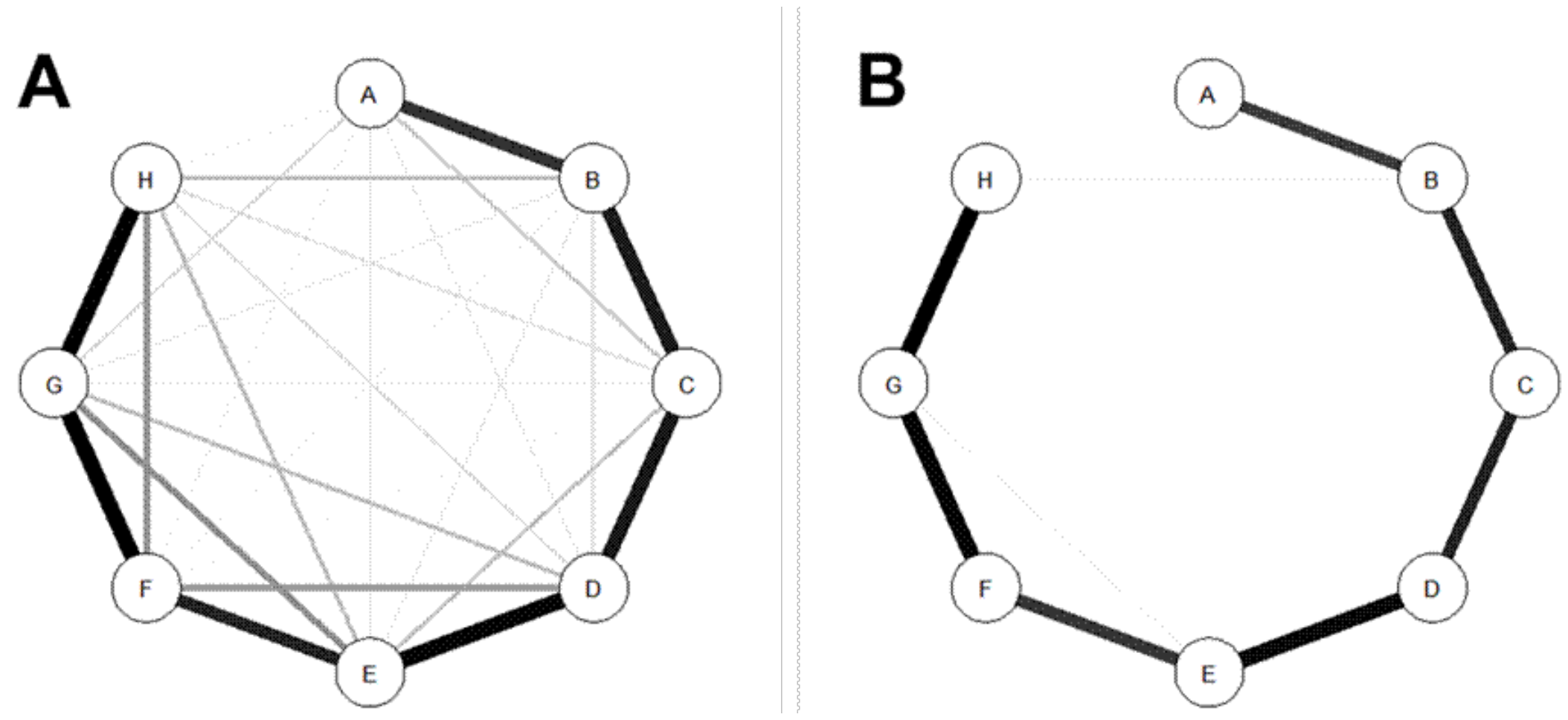

Note. Panel A shows the network based on pairwise correlations among variables A–H, where edges reflect both direct and indirect associations. Panel B shows the partial correlation network estimated by the EBICglasso function, where edges represent conditional dependencies controlling for all other variables.

Section I: Weighted Results

Studies leveraging psychological networks mostly did not leverage weights (e.g., Fishman & Davis, 2021). Still, as a robustness check, we re-estimated all models using the weighted version of the EVS/WVS dataset. The weight variable (*gwght*) is designed to adjust the sample distribution to better reflect the socio-demographic composition of the target population. Specifically, the weights correct for differences in age, sex, educational level, and region of residence between the survey sample and the population benchmarks (see EVS 2017 Weighting data [http://0.21241/ssoar.70113] and WVS documentation [https://www.worldvaluessurvey.org/WVSContents.jsp?CMSID=WEIGHT&CMSID=WEIGHT]). Applying these weights ensures that our analyses are not disproportionately influenced by over- or under-represented groups in the sample.

Generally, the results obtained with the weighted data are consistent with those reported in the main text. We find that disagreement is still negatively correlated with symbolic-operational ideological alignment (Spearman's $\rho = -.280$, $p = .013$) and within-operational ideological alignment (Spearman's $\rho = -.104$, $p = .363$), though the latter is insignificant. Within-operational ideological alignment and symbolic-operational ideological alignment remain strongly positively correlated (Spearman's $\rho = .501$, $p < .001$). The following three figures (Figure I1-I2, Table I1) also shows the global distribution and major regression results are not influenced by the weighting issue.

Figure I1 *Scatter plots between each pair of manifestations of mass polarization (weighted)*

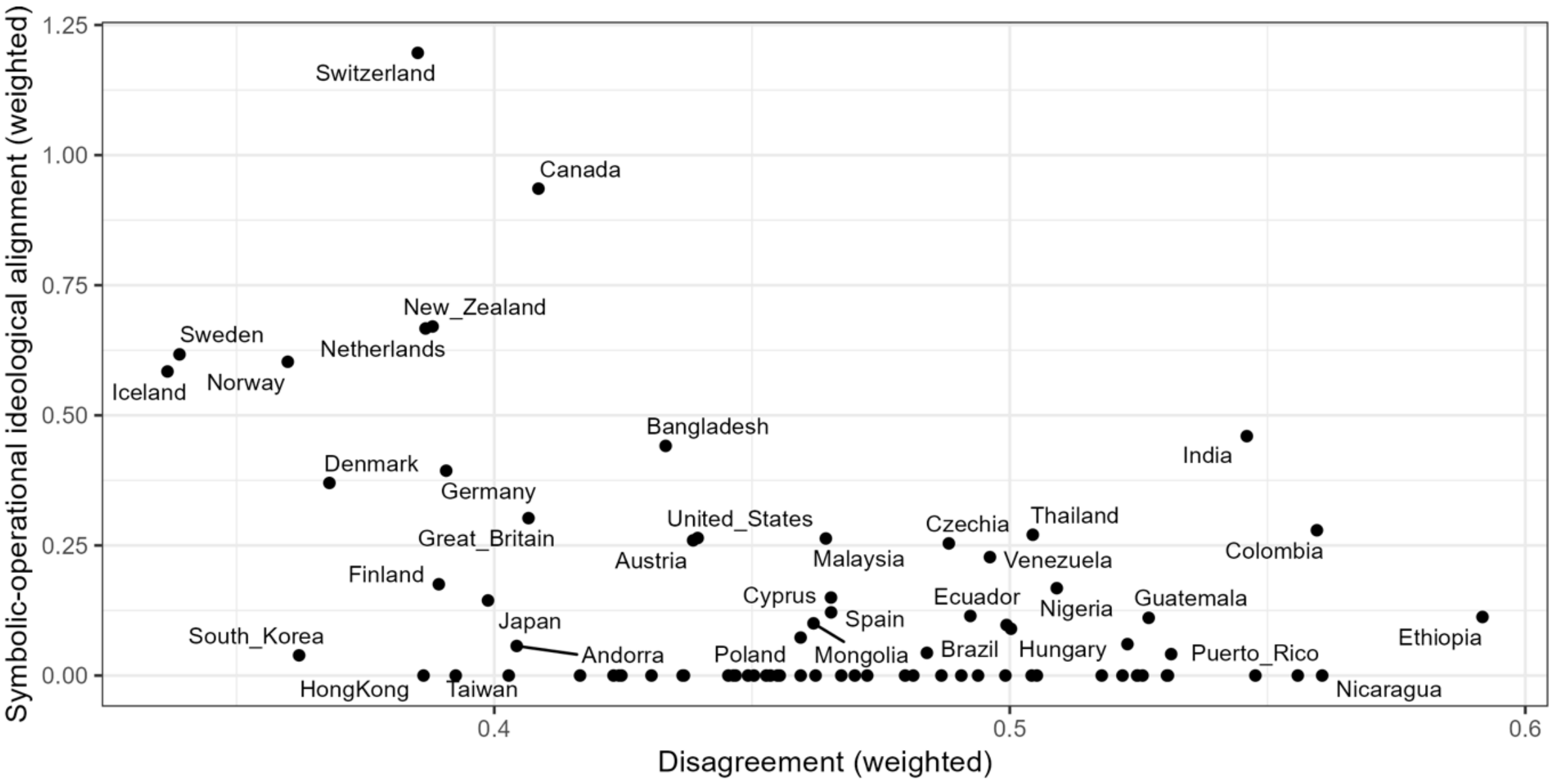
Symbolic-operational ideological alignment (weighted)
1.25
1.00
0.75
0.50
0.25
0.00
Switzerland
Canada
New_Zealand
Sweden
Netherlands
Norway
Iceland
Bangladesh
India
Denmark
Germany
United_States
Great_Britain
Czechia
Thailand
Austria
Malaysia
Venezuela
Colombia
Finland
Cyprus
Ecuador
Nigeria
Guatemala
Japan
Spain
Ethiopia
South_Korea
Andorra
Poland
Mongolia
Brazil
Hungary
Puerto_Rico
HongKong
Taiwan
Nicaragua
0.4
0.5
0.6
Disagreement (weighted)

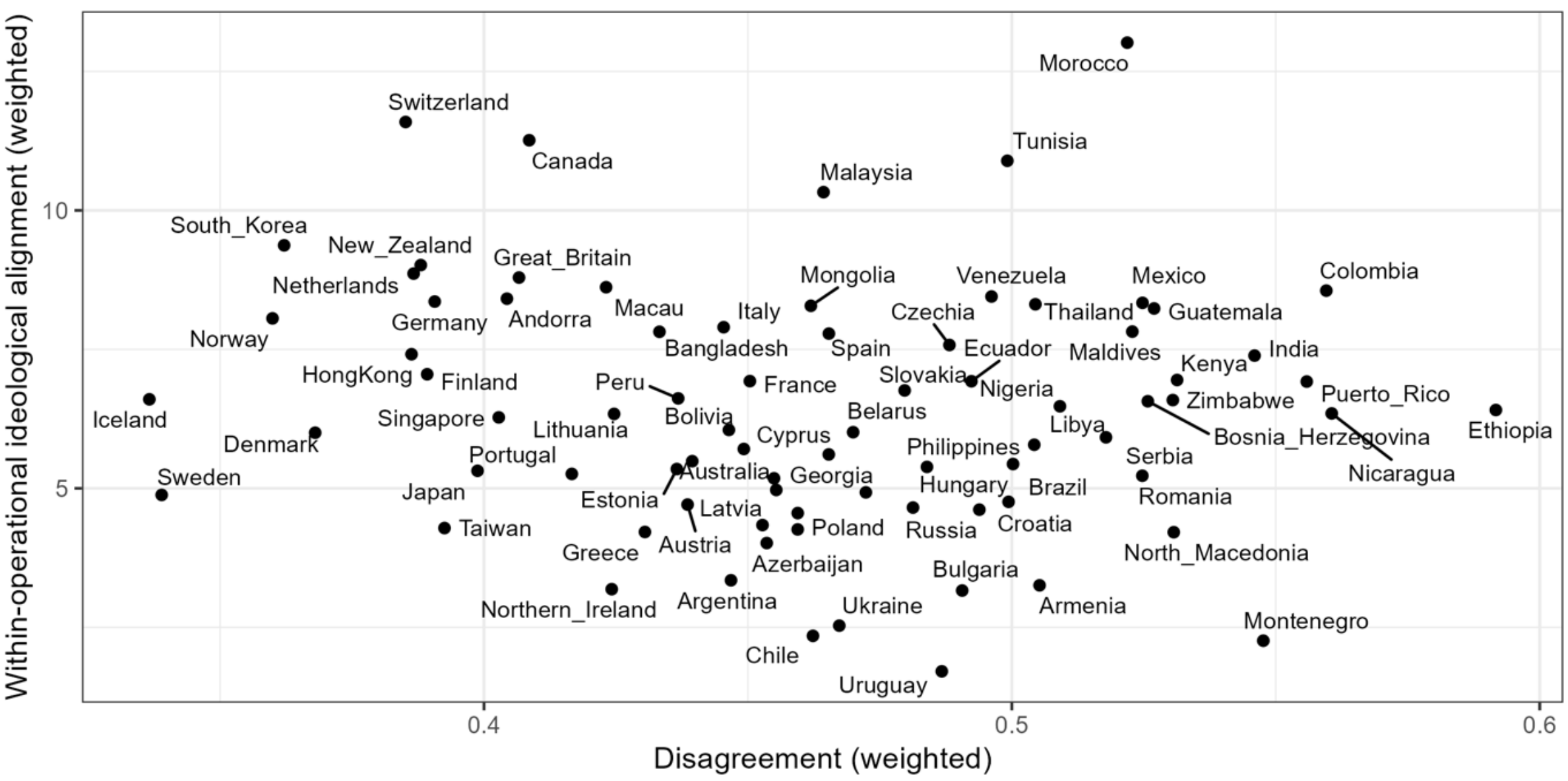
Within-operational ideological alignment (weighted)
10
5
Morocco
Switzerland
Tunisia
Canada
Malaysia
South_Korea
New_Zealand
Great_Britain
Netherlands
Mongolia
Venezuela
Mexico
Colombia
Macau
Italy
Czechia
Thailand
Guatemala
Germany
Andorra
Norway
Bangladesh
Spain
Ecuador
Maldives
India
HongKong
Finland
Peru
France
Slovakia
Nigeria
Kenya
Puerto_Rico
Iceland
Singapore
Bolivia
Belarus
Zimbabwe
Ethiopia
Lithuania
Cyprus
Libya
Bosnia_Herzegovina
Denmark
Portugal
Philippines
Serbia
Sweden
Australia
Georgia
Hungary
Brazil
Nicaragua
Japan
Estonia
Latvia
Romania
Taiwan
Poland
Russia
Croatia
Greece
Austria
North_Macedonia
Azerbaijan
Bulgaria
Northern_Ireland
Argentina
Armenia
Ukraine
Montenegro
Chile
Uruguay
0.4
0.5
0.6
Disagreement (weighted)

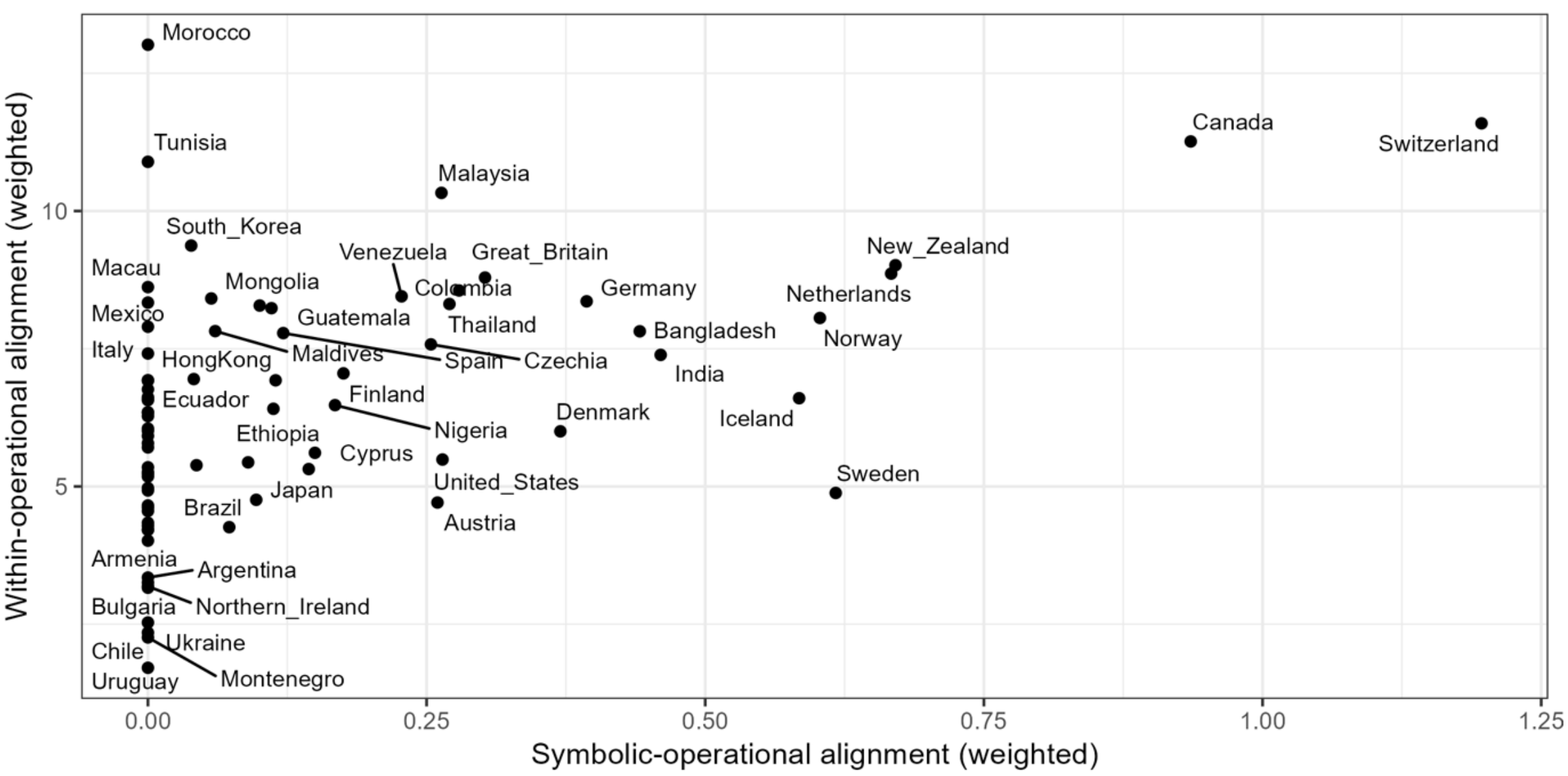
Within-operational alignment (weighted)
10
5
Morocco
Tunisia
Malaysia
Canada
Switzerland
South_Korea
New_Zealand
Macau
Venezuela
Great_Britain
Mongolia
Colombia
Germany
Netherlands
Mexico
Guatemala
Thailand
Bangladesh
Norway
Italy
HongKong
Maldives
Spain
Czechia
India
Ecuador
Finland
Denmark
Iceland
Ethiopia
Nigeria
Cyprus
Sweden
United_States
Japan
Brazil
Austria
Armenia
Argentina
Bulgaria
Northern_Ireland
Ukraine
Chile
Uruguay
Montenegro
0.00
0.25
0.50
0.75
1.00
1.25
Symbolic-operational alignment (weighted)

Figure I2 *Regression coefficients of OLS models predicting mass ideological polarization (weighted)*

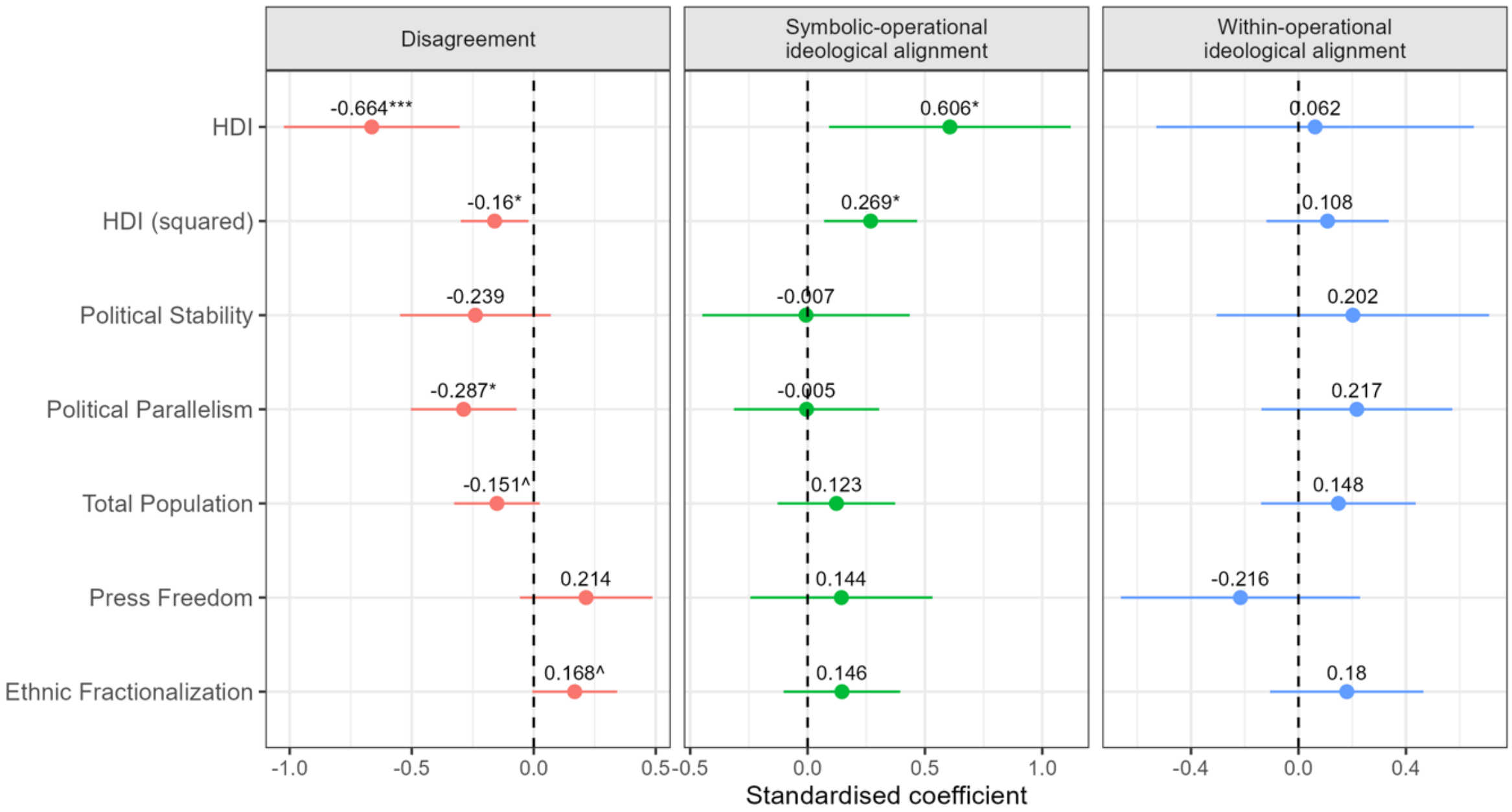

Note. Standardized coefficients are reported, and bars represent 95% confidence intervals.
$^{*}p < .05$, $^{**}p < .01$, $^{***}p < .001$.

Table I1 *OLS Models Predicting Disagreement (weighted)*

	Dependent Variable: Disagreement			
	(1)	(2)	(3)	(4)
Symbolic-operational ideological alignment	-0.482^{***}	$-.178$		
	(0.109)	(0.090)		
Within-operational ideological alignment			-0.068	-0.047
			(0.124)	(0.080)
HDI		-0.807^{***}		-0.917^{***}
		(0.111)		(0.097)
HDI-squared		-0.136		-0.189^{**}
		(0.068)		(0.064)
Adj R^2	.220	.618	.011	.597
N	67	67	67	67

Note. Unstandardized coefficients with standard errors in parentheses.
$^{*}p < .05$, $^{**}p < .01$, $^{***}p < .001$.

References

Coppedge, Michael, John Gerring, Carl Henrik Knutsen, Staffan I. Lindberg, Jan Teorell, David Altman, Fabio Angiolillo, Michael Bernhard, Cecilia Borella, Agnes Cornell, M. Steven Fish, Linnea Fox, Lisa Gastaldi, Haakon Gjerløw, Adam Glynn, Ana Good God, Sandra Grahn, Allen Hicken, Katrin Kinzelbach, Kyle L. Marquardt, Kelly McMann, Valeriya Mechkova, Anja Neundorf, Pamela Paxton, Daniel Pemstein, Oskar Rydén, Johannes von Römer, Brigitte Seim, Rachel Sigman, Svend-Erik Skaaning, Jeffrey Staton, Aksel Sundström, Eitan Tzelgov, Luca Uberti, Yi-ting Wang, Tore Wig, and Daniel Ziblatt. 2024. "V-Dem Codebook v14" Varieties of Democracy (V-Dem) Project.

Drazanova, L. (2019). Historical index of ethnic fractionalisation dataset. *Harvard Dataverse*. https://dataverse.harvard.edu/dataset.xhtml?persistentId=doi:10.7910/DVN/4JQRCL

Fishman, N., & Davis, N. T. (2022). Change we can believe in: Structural and content dynamics within belief networks. *American Journal of Political Science*, *66*(3), 648–663.

Jost, J. T., Federico, C. M., & Napier, J. L. (2009). Political ideology: Its structure, functions, and elective affinities. *Annual Review of Psychology*, 60(1), 307-337.

Keskintürk, T. (2022). The organization of political belief networks: a cross-country analysis. *Social Science Research*, 107, 102742.

Mehlhaff, I. D. (2025). *Mass Polarization across Time and Space*. Cambridge University Press.

Pan, J., & Xu, Y. (2018). China's ideological spectrum. *The Journal of Politics*, *80*(1), 254-273.

Solt, F. (2020). Measuring Income Inequality Across Countries and Over Time: The Standardized World Income Inequality Database. Social Science Quarterly 101(3):1183-1199. SWIID Version 9.9

Zechmeister, E. J., & Corral, M. (2013). Individual and contextual constraint on ideological labels in Latin America. *Comparative Political Studies*, *46*(6), 675-701.
